# Selective Release of MicroRNA Species from Normal and Malignant Mammary Epithelial Cells

**DOI:** 10.1371/journal.pone.0013515

**Published:** 2010-10-20

**Authors:** Lucy Pigati, Sree C. S. Yaddanapudi, Ravi Iyengar, Dong-Ja Kim, Steven A. Hearn, David Danforth, Michelle L. Hastings, Dominik M. Duelli

**Affiliations:** 1 Department of Pathology, The Chicago Medical School, Rosalind Franklin University of Medicine and Science, North Chicago, Illinois, United States of America; 2 Department of Cell Biology and Anatomy, The Chicago Medical School, Rosalind Franklin University of Medicine and Science, North Chicago, Illinois, United States of America; 3 The Bligh Cancer Research Center, The Chicago Medical School, Rosalind Franklin University of Medicine and Science, North Chicago, Illinois, United States of America; 4 Cold Spring Harbor Laboratory Microscopy Facility, Cold Spring Harbor Laboratory, Cold Spring Harbor, New York, United States of America; 5 Surgery Branch, Center for Cancer Research (CCR), National Cancer Institute (NCI), National Institutes of Health (NIH), Bethesda, Maryland, United States of America; INSERM U1016, Institut Cochin, France

## Abstract

MicroRNAs (miRNAs) in body fluids are candidate diagnostics for a variety of conditions and diseases, including breast cancer. One premise for using extracellular miRNAs to diagnose disease is the notion that the abundance of the miRNAs in body fluids reflects their abundance in the abnormal cells causing the disease. As a result, the search for such diagnostics in body fluids has focused on miRNAs that are abundant in the cells of origin. Here we report that released miRNAs do not necessarily reflect the abundance of miRNA in the cell of origin. We find that release of miRNAs from cells into blood, milk and ductal fluids is selective and that the selection of released miRNAs may correlate with malignancy. In particular, the bulk of miR-451 and miR-1246 produced by malignant mammary epithelial cells was released, but the majority of these miRNAs produced by non-malignant mammary epithelial cells was retained. Our findings suggest the existence of a cellular selection mechanism for miRNA release and indicate that the extracellular and cellular miRNA profiles differ. This selective release of miRNAs is an important consideration for the identification of circulating miRNAs as biomarkers of disease.

## Introduction

MicroRNAs (miRNAs) are small RNA molecules that are defined by structure, regulatory functions, and mode of biogenesis. In a canonical pathway, miRNAs are produced as primary miRNA transcripts (pri-miRNA), which are processed by the Microprocessor complex that includes Drosha into pre-miRNA molecules. Pre-miRNAs are exported from the nucleus, and further processed by Dicer to yield mature miRNAs that associate with the RNA-Induced Silencing Complex, RISC and target mRNA. Changes in the abundance of miRNAs have been documented in various diseases including malignancies such as breast cancer. The cellular miRNA composition has been explored for diagnosis and prognosis of breast cancer and other diseases [1], [2], [3], [4], [5], [6], [7], [8], [9], [10]. Because upregulated miRNAs of lymphoma, prostate, lung and breast cancers have also been detected in blood plasma and serum [11], [12], [13], [14], circulating miRNAs are currently evaluated as surrogate biomarkers for breast cancer [14], [15], [16], [17], other cancers [13], [18], [19], [20], diseases or conditions [21].

Cells in culture and in the body release a variety of nucleic acids into the environment, including cellular and viral mRNAs and miRNAs. At least some of these RNAs have been found to be encapsulated in micro- and nano-vesicles released from cells [22], [23], [24], [25], [26], [27], [28]. One such vesicle is the exosome, which originates from multivesicular bodies (MVB), and is released by cells in the body and in culture [23], [24], [25], [27], [29]. For ovarian and lung cancer and glioblastoma, circulating miRNAs have been found present in exosome-like vesicles [19], [20], [30].

While mechanisms that control the release of viral RNA have been studied extensively, the mechanism of miRNA release is not clearly understood. Recent data suggest that miRNA release may occur through a ceramide-dependent secretory machinery [31]. In addition, mature miRNAs have been reported to be released from cells in exosomes as a consequence of the MVB's role in loading miRNAs to their complementary target mRNA in the RNA-Induced Silencing Complex, RISC [32]. However, immature miRNAs are also released [29] and apoptotic bodies are thought to contain miRNA [33], suggesting alternative mechanisms of miRNA release.

Blood contains vesicular miRNA of many cells, which may make it necessary to enrich organ, tissue, or cell-type-specific exosomes using surface markers for proper quantitation [19], [20], [34]. However, in addition to releasing miRNAs into blood, mammary epithelia produce and condition specialized body fluids, including mammary fluid in the resting gland, and milk during lactation. Thus, mammary fluids might provide an alternate body fluid to measure diagnostic miRNAs of extracellular human mammary epithelial cell (HMEC) for breast disease.

For identification of circulating diagnostic miRNAs, most approaches thus far have focused on quantifying circulating miRNAs that are overexpressed or lost in the cancer cell of origin [13], [18], [30], [35]. However, only some of the highly abundant cellular miRNAs have been found in higher concentrations in circulation, suggesting that only a subset of cellular miRNAs are released into the environment [29]. These findings prompted us to test whether cells release different miRNAs than they retain. We found that nearly 30% of the released miRNAs *in vitro* and *in vivo* do not reflect the cellular profile, suggesting that some miRNAs are retained or released selectively. Some selectively released miRNAs were enriched in body fluids conditioned by mammary cells, including mammary fluids, blood and milk. This subset of miRNAs may have value in breast cancer diagnosis and biology. Our results stress that miRNAs are released selectively, and that extracellular miRNAs should be considered independent of cellular miRNAs abundance when considering diagnostic markers of disease.

## Results

### Mammary epithelial cells release exosomal vesicles with a distinct small RNA profile

**Figure 1:**
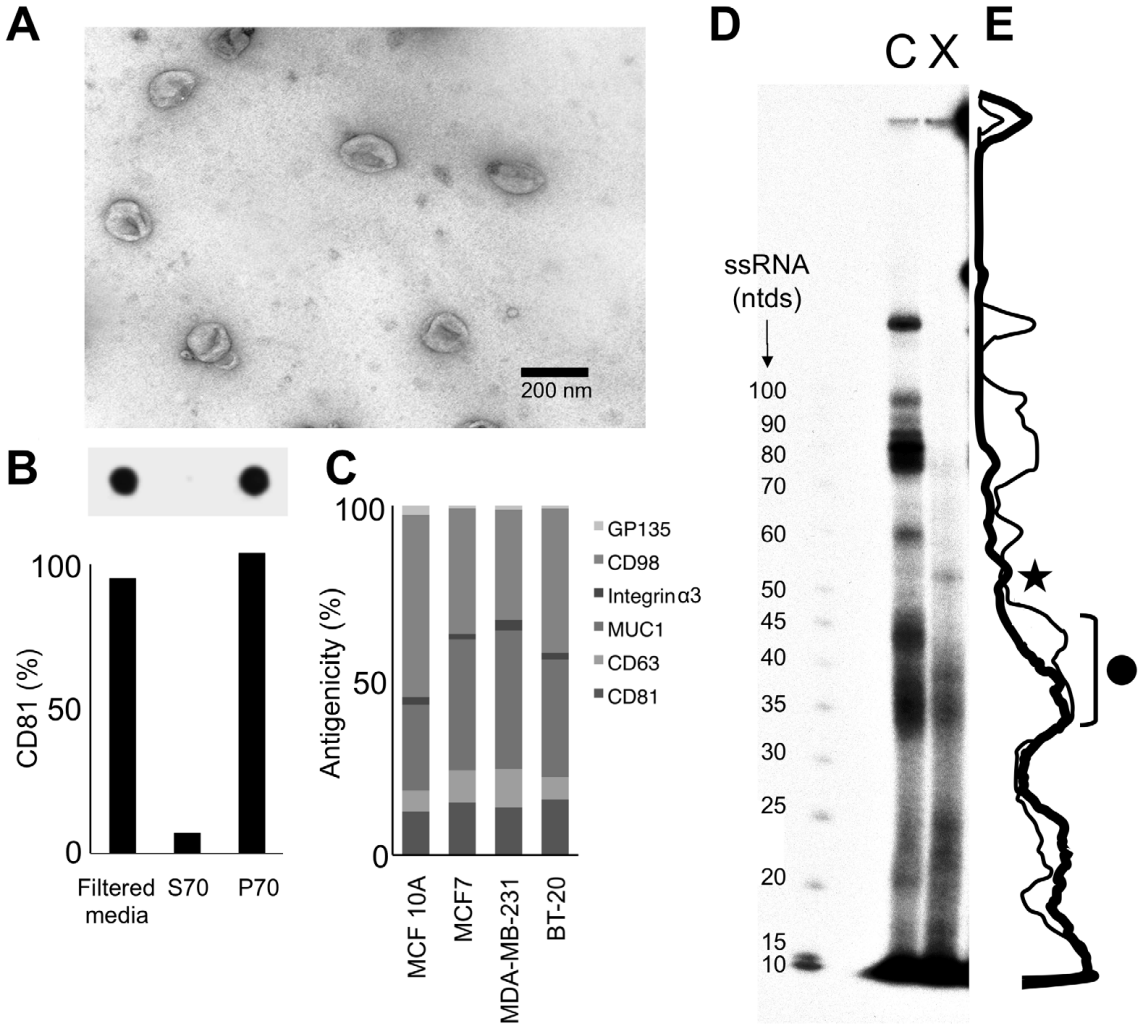
Differential Cellular Release and Retention of Small RNAs. Medium conditioned by MCF7 cells for 5 days was enriched for exosomes by a filtration and ultracentrifugation protocol producing a P70 preparation. **A** The P70 was subjected to negative-staining EM. **B** The abundance of tetraspanin CD81, an exosome-marker was assessed in the filtered conditioned medium, the P70 pellet obtained by ultracentrifugation, and the supernatant (S70) using slot-blot (inset, n = 2). **C** The surface antigens CD81, CD63 and Mucin-1 were detected in the P70 fraction of the mammary epithelial cells using slot-blot. The absolute amount of bound antibody was quantified using standard-curves of antibody dilutions, and expressed as a percent of total antigenicity for the P70 of each cell line. The data of two replicate experiments for the indicated cell lines are shown. **D** Radiolabeled small RNAs isolated from MCF7 cells (c) and the extracellular preparation P70 (x) were separated by PAGE on a 12% denaturing gel. **Star**: Extracellular enriched RNA; **Circle**: Some extracellular RNAs identified by sequencing (see text and **Tables S1** and **S2**). **E** Quantitation of labeled RNA species of D. The thin line indicates abundance of cellular small RNAs, whereas the thick line indicates the abundance of the extracellular miRNAs.

To compare intracellular and extracellular miRNA populations, we analyzed the breast cancer cell line MCF7, which releases exosomes [36]. We focused on RNA contained in vesicles, because such vesicles are released from cells *in vitro*, as well as into blood, urine, saliva and other body fluids [14], [15], [19], [20], [23], [24], [25], [27], [30], [37]. We enriched for vesicles by centrifugation at 70,000 g from media conditioned by MCF7 cells (**Figure 1**). This preparation (P70) included cup-shaped vesicles of about 100 nm (**Figure 1A**), which is consistent with exosomes; and was enriched in CD81, a marker protein of exosomes (**Figure 1B**).

To assess if other human mammary epithelial cells release exosomes, we quantified the abundance of CD81 and CD63, an endosomal marker protein, in P70 preparations of breast cancer cell lines MDA-MB-231, BT-20, and the nontumorigenic mammary epithelial cell line MCF 10A. We found that P70 preparations of all cell lines contained CD63 and CD81 at similar proportions as MCF7 cells (**Figure 1C**), suggesting that mammary epithelial cells in general release similar vesicles.

We also tested for the presence of the epithelial-cell marker Mucin-1 and other antigens on vesicles released from mammary epithelial cells (**Figures 1C**). We found several proteins with roles in mammary biology present in similar abundance, leading us to conclude that vesicles of mammary epithelial cells share antigenic properties.

To compare the cellular to the extracellular small RNA population, we collected total RNA of MCF7 cells or media conditioned by these cells after 5-days of culturing. We then radiolabeled small RNAs enriched from MCF7 cells (c) and P70 (x) and analyzed their migration by polyacrylamide gel electrophoresis (PAGE) and autoradiography (**Figures 1D and 1E**).

We found that the majority of small RNAs migrated differently in extracellular than intracellular preparations, indicating that the released and retained RNA populations were not the same. Furthermore, the extracellular fraction included some RNA species that were less abundant in the cell. For example, in one band that is highly enriched in the P70, we identified by sequencing, RNAs that are cleavage products of 5.8S rRNA and a U1 small nuclear RNA (**Figure 1E**, and **Table S1A and S1B**), whereas common RNAs of a region with a similar banding pattern in cellular and extracellular preparations contained 28S rRNA fragments (**Table S2**). Therefore the extracellular RNA population is enriched in some RNA species underrepresented in the cell.

### Extracellular and cellular miRNA populations are different

**Figure 2:**
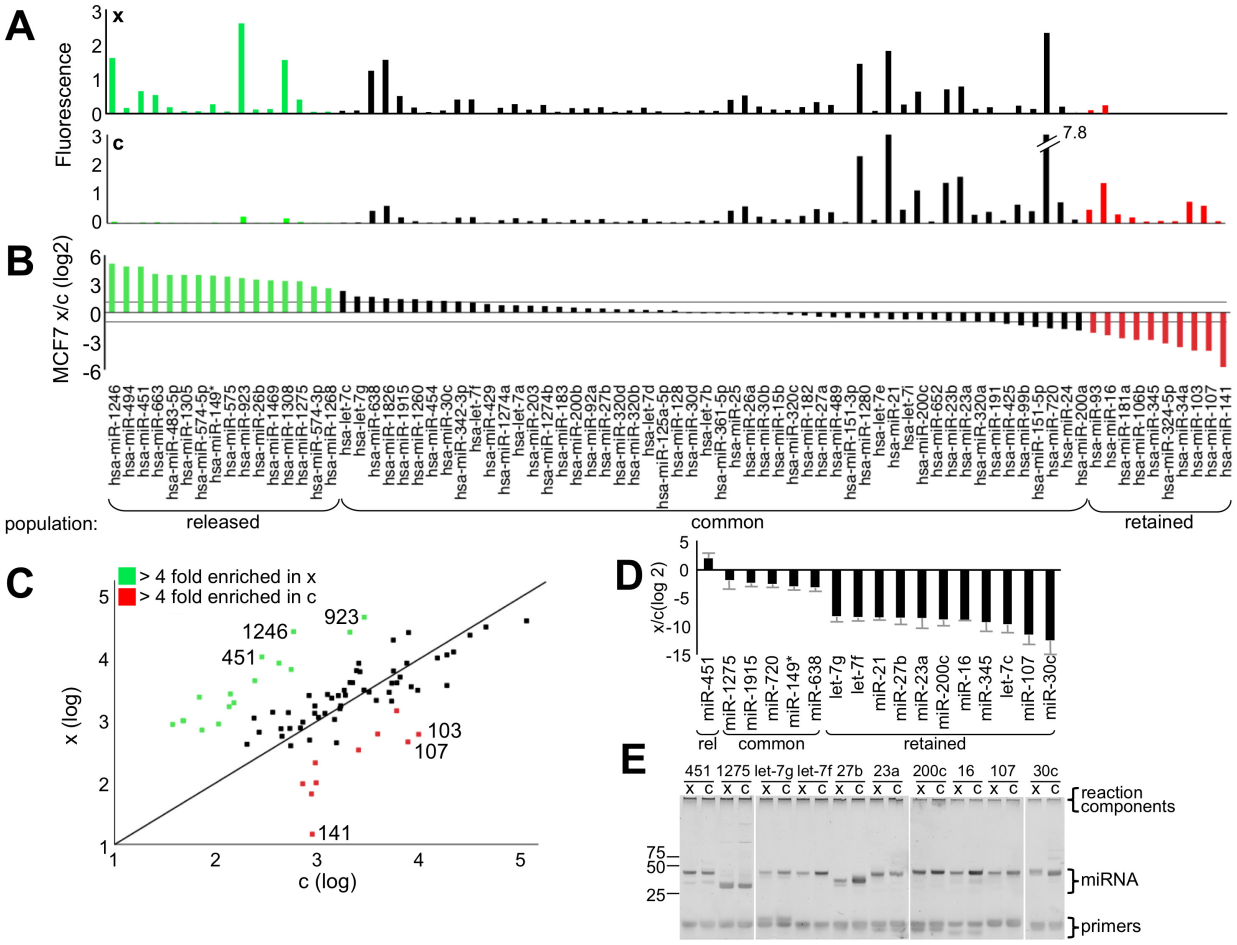
Some MicroRNAs are Released Disproportionately. Duplicate microRNA microarrays were hybridized with 1 µg of total cellular or 1 µg of extracellular miRNA from MCF7 cells. Results are plotted as **A** relative fluorescent intensities of extracellular (x, upper panel) and cellular (c, lower panel) miRNAs, or **B** ratio of extracellular to cellular miRNAs. The horizontal lines in B indicate the threshold of 2 fold-changes, whereas the red and the green marked populations indicate a greater than 4-fold enrichment in the released extracellular (A, upper panel, x), or in the cells (A, lower panel, c) respectively. **C** Scatter plot of average reads of the miRNAs quantitated by array. Only miRNAs with a fluorescent value of greater than 500 in the cellular or extracellular population are shown (see Materials and Methods). The numbers next to dots indicate the miRNA the dot represents. **D** MCF7 cells were cultured for 5 days, and the total amount of specific cellular and extracellular miRNAs were measured by quantitative linker-ligation mediated RT-PCR, and the miRNA ratios were plotted. The average of 3 independent experiments is shown. **E** Native PAGE of products at end-point of quantitative RT-PCR. The major PCR-products between 32–48 ntds correspond to the mature miRNA (miRNA) as expected by size and determined by sequencing (**Table S5**). The bands with a migration of less then 25 ntds are the PCR primers (primers) used in the reaction. Bands that retained in the well are amplification-independent reaction components (reaction components). Hsa-miR-923 has since been reclassified as a specific rRNA fragment.

Because of the suggested roles of extracellular miRNAs in signaling and diagnosis [27], we investigated whether the intracellular and extracellular miRNA composition are the same. To answer this question, we performed microRNA microarray analyses of MCF7 cellular (c) and extracellular (x) RNAs (**Figure 2A**), and found that about 66% of the released miRNAs are at an abundance that closely reflects the cellular miRNA abundance (**Figures 2B and 2C**). This finding is in agreement with a model wherein most, but not all miRNAs are released passively by mass action.

To confirm the miRNA composition determined by microarray hybridization, we measured the cellular and extracellular mature miRNA populations by quantitative PCR approaches after reverse-transcription of miRNAs (qRT-PCR). We used two approaches that are specific for mature human miRNAs: 3′ linkering as modified from [38], and stem-loop primers [39] (**Tables S3 and S4**). We first focused on miRNAs that were reported to be involved in mammary and cancer biology or cancer diagnostics [1], [6], [7], [15], [40], [41], [42], [43], [44], [45], [46] (**Figure 2D and E**). We verified the identity of many species by sequencing (**Table S5**). The quantified miRNAs were normalized to a synthetic RNA (INT-RNA) to control for RNA recovery (**Table S4** and Materials and Methods).

Absolute quantitation of miRNAs allowed us to determine that only a small portion of the cellular pool of most miRNA species is released. In particular, only about 2% of the most abundant miRNA, miR-720 was released into the environment within 5 days of culturing (**Figure 2D**, and data not shown). Importantly, we confirmed that many miRNAs were represented at comparable proportions in the cellular and extracellular population (e.g. miR-638), whereas several other miRNA species were overrepresented either of these populations (e.g. miR-451 and miR-107) (**Figure 2D**). These data suggest that cells have a mechanism in place to select some miRNAs for cellular release or retention. Therefore, we investigated the selective nature of miRNA release further.

### miR-16 is a surrogate marker of bulk exosome release

**Figure 3:**
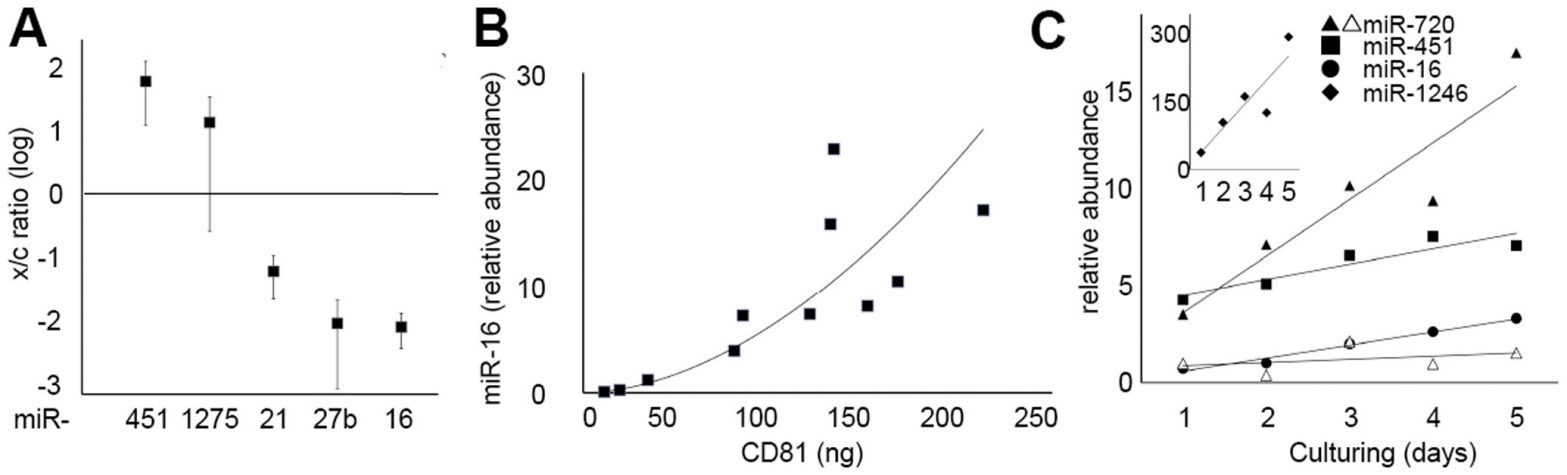
Extracellular miR-16 Levels Correlate with Nanovesicle Abundance. **A** Plot of extracellular to intracellular levels of miRNAs quantitated by qRT-PCR using stem-loop primers of cells lines BT-20, MCF 10A, MCF7, MDA-MB-231 and SK-BR-3. MiRNA levels were calculated using standard curves, and corrected for recovery by normalization to a spiked synthetic RNA (INT-RNA) introduced at the time of RNA-extraction (Materials and Methods and Table S4). Error bars indicate the measure of one standard deviation of 3 independent experiments. **B** Relative levels of miR-16 and absolute levels of CD81 were measured in P70 fractions of seven independent experiments and plotted. The line indicates a best-fit power curve (r^2^ = 0.95). **C** The P70 was collected daily from media conditioned by MCF7 cells (solid symbols), or from MCF 10A cells (open symbol) cultures and miRNAs were quantified in triplicate. The release rates were linear; r^2^ = 0.79 for miR-1246 (inset), r^2^ = 0.98 for miR-16, r^2^ = 0.85 for miR-451; and r^2^ = 0.87 for miR-720 of MCF7 cells.

Because the transformation status of a cell regulates exosome secretion [47], and thus possibly exosomal miRNA release, we sought to identify a miRNA that faithfully reflects exosomal abundance in order to quantify selective miRNA release into vesicles. For example, miR-103 and its paralog miR-107 have high and consistent expression in both cancerous and normal mammary tissues, and thus have been used for normalizing miRNA studies comparing mammary cells [42]. However, these miRNAs were grossly underrepresented in the extracellular fraction (**Figures 2 A and B**), highlighting the observation that at least some miRNAs with diagnostic value in cells are not represented in the released population. We therefore considered other miRNAs for normalization. Using the more sensitive stem-loop primer PCR approach [39], we focused on several miRNAs, including the most abundantly released miRNA of MCF7 cells, miR-720; miR-21; miR-16; and microRNAs that were enriched extracellularly, including miR-451, miR-1275 (**Figure 3A**). We measured the cellular to extracellular ratio of these miRNAs in a set of HMECs including cell lines BT-20, MCF 10A, MCF7, MDA-MB-231, and SK-BR-3. We found that HMECs irrespective of malignancy, released a constant amount of miR-21 reflective of cellular abundance, a miRNA that is upregulated in many breast cancers [6], [7], [8], and miR-16 (**Figure 3A**). The most consistently released miRNA was miR-16 of which for each released molecule, 160–400 molecules were retained.

We tested whether exosome release correlated with miR-16 release, and found that the abundance of extracellular miR-16 correlated with the amount of microvesicular marker protein CD81 released from cells (**Figure 3B**). This observation suggested that miR-16 is not subject to a selection mechanism beyond packaging into exosomes.

The consistency of release, and the fact that miRNA-16 is known to have relatively high and stable expression in both normal and transformed breast tissue [48], led us to conclude that extracellular miR-16 is a surrogate marker for the abundance of vesicular miRNAs. Using miR-16 as an internal standard, we addressed the observed differences in the release rate of specific miRNAs.

### Extracellular miRNA accumulation is linear

We measured properties of extracellular miRNAs, including their release rate and stability. To do so, we focused on miR-16, miR-1246, miR-451 and miR-720. We found that individual miRNAs were released at different rates. MiR-16 accumulated the slowest, with less than a doubling of the extracellular miRNA concentration per day. In contrast, the extracellular accumulation of miR-1246 more than doubled per day (**Figure 3C**). These rates were also different among cell lines. For example the rate of accumulation of miR-720 was about 17 times higher in MCF7 cells than MCF 10A cells (**Figure 3C**). However, we found that the concentrations of these miRNAs dropped no more than 2-fold upon storage of P70 for up to 3 days, the maximum time we measured (data not shown), suggesting that stability alone cannot account for the differences in extracellular miRNA levels. These findings fit with a model wherein miRNAs are released at a constant and miRNA-species specific rate.

### Some diagnostic miRNAs are selectively retained by cells

Our array analyses indicated that 13% of the MCF7 miRNA species were selectively retained by the cell. This category included miR-141, which was nearly undetectable in the released population (**Figures 2A and 2B**), and which has roles in carcinogenesis [**49**]. In contrast, miR-141 is abundantly released from other malignancies, including ovarian [50] and prostate cancer cells [13], [19]. Therefore, retention of some miRNAs is cell-type specific.

**Figure 4:**
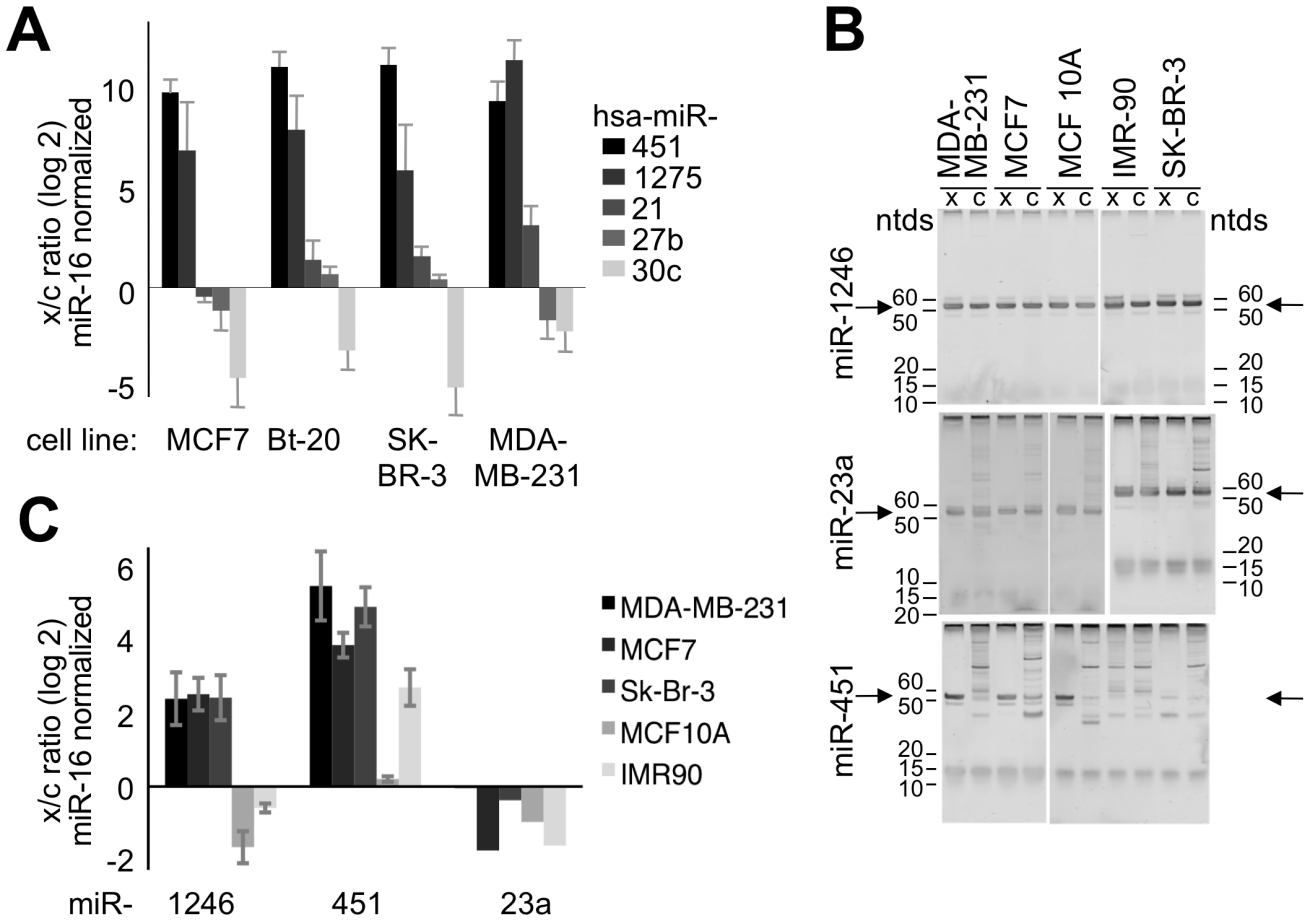
miR-451 and miR-1246 are Selectively Released from Cells. **A** The ratio of released to retained miRNAs standardized to released and retained levels of miR-16 respectively, was quantified from the indicated cell lines using the linker-ligation qRT-PCR approach. **B** Native PAGE of products at end-point of quantitative RT-PCR of indicated miRNAs using the stem-loop primer approach, and quantified in **C**. The major PCR-products, indicated by arrows, correspond to the mature miRNA as expected by size (53 ntds for miR-1246, 53 ntds for miR-23a, and 56 ntds for miR-451) and determined by sequencing (**Table S5**). The bands with a migration of less then 15 ntds are the PCR primers used in the reaction. The stained material in the well is amplification-independent material of the reaction kit. The minor bands larger than 60 ntds represent artifactual products amplified at low template concentration (**Figure S5**). All miRNAs were quantified from at least 3 cell and media collections, except for miR-23a, which was measured only twice. Error bars indicate standard deviation.

To test whether the retention of other miRNAs was cell-type and miRNA specific, we quantified the release of miR-27b, miR-30c, and miR-23a from several cell lines using qRT-PCR. We found that for these miRNA species, for each 100 molecules produced, 0.5–20 molecules were released by most but not all breast cancer cell lines tested (**Figures 2A, 2D** **3A** **and** **Figure 4**). This suggests, that in general, the retention of specific miRNAs is a common phenomenon for breast cancer cells.

We tested if miRNAs with established roles in mammary biology were also selectively retained by cells. We tested let-7c, with roles in Myc regulation (reviewed in [45]) and in HMEC progenitor cells [51]; miR-99a, which clusters with let-7c, and resides in a commonly deleted chromosome region of lung [52] and primary breast cancer [53]; miR-196a1, which is overexpressed in breast cancer cells [54], miR-210, a hypoxia sensor with prognostic value in breast cancer [1], [54], [55]; miR-200b, a regulator of epithelial-mesenchymal transition (EMT), [41], [56], [57]; and several miRNAs associated with metastasis, miR-148a [58], miR-335[5], miR-373 and miR-520c [3]). We found all of these miRNAs in the MCF7 cellular component, yet we detected none (**Figure S1A**) or very few (**Figure S1B**) of these miRNAs in the extracellular population. Another miRNA, miR-1275, ranged from 5-fold higher retention in MDA-MB-231 cells to 3-fold enrichment in the spent media of MCF 10A, BT-20, and SK-BR-3 cells (**Figures 2D** **,** **Figure 3** **, and** **Figures 4A and 4B**). Therefore miR-1275 release did not correlate with the transformation status of its cell of origin. Our findings that some miRNAs were underrepresented in the extracellular population might explain reports in which increased plasma and serum levels of some cancer-associated miRNAs, including oncogenic miR-155, or miR-21 [**1**], [54****], could not be found in all breast cancer patients [15], [17]. Furthermore, this finding raises the possibility that cells have mechanisms in place that retain miRNAs with roles in carcinoma growth [59], cell differentiation [43], [60], [61] and metabolism [62].

### Selective release of immature miRNAs

**Figure 5:**
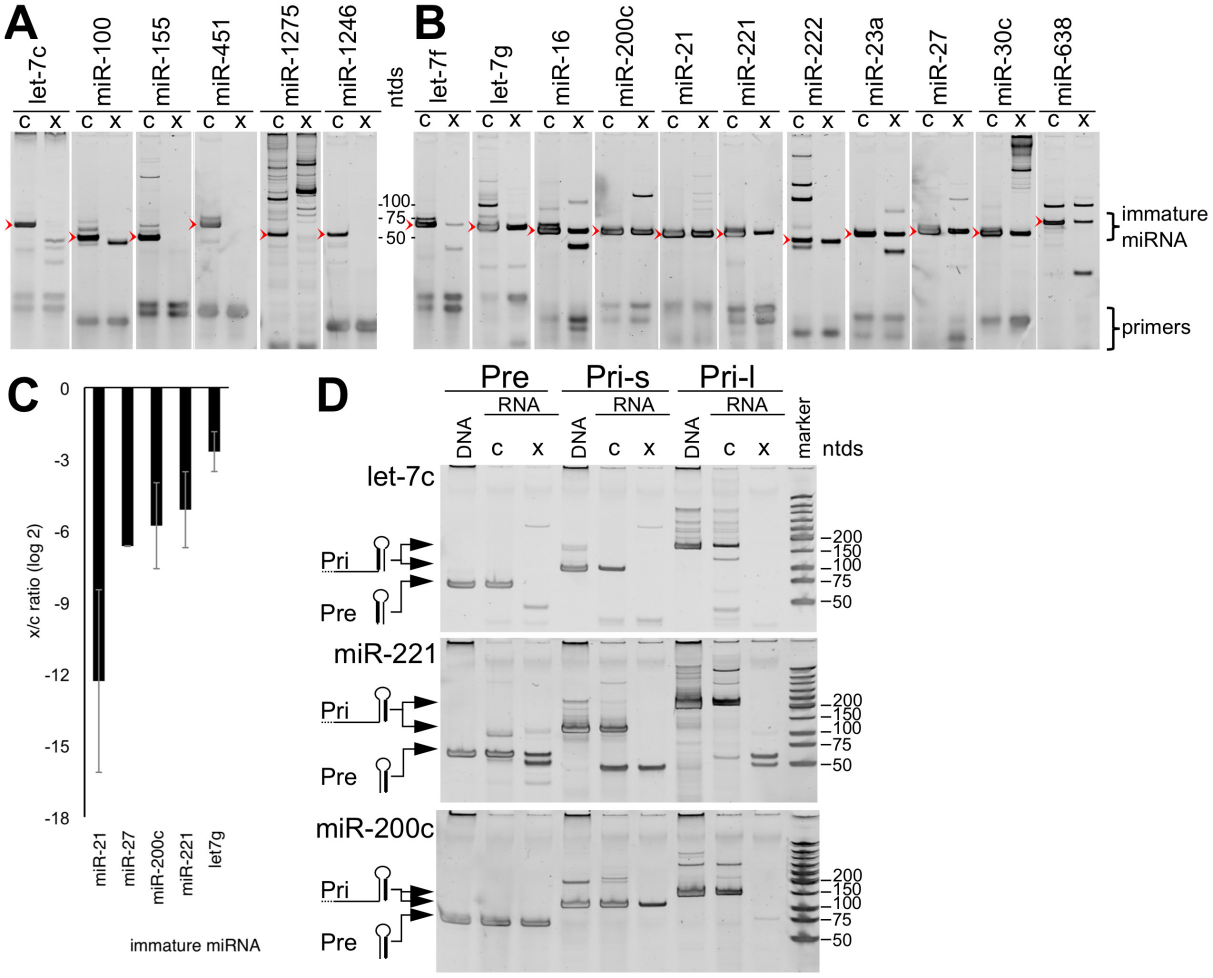
Immature miRNAs are Released at miRNA-Species Specific Rates. **A** and **B**, pre-miRNAs of MCF7 cells (c), and miRNAs released from these cells (x) were amplified by rt-PCR and subjected to native PAGE. All the main bands correspond to the expected size of the amplification products of immature miRNAs (arrowheads). Some of the amplification products were confirmed by sequencing (**Table S7**). **C** Those miRNAs that yielded single bands in 3 independent experiments were quantified by qRT-PCR. **D** Presence of upstream RNA sequences, corresponding to pri-miRNA sequences (Pri-s and Pri-l), and RNA corresponding to pre-miRNAs (Pre) were assed by PCR. The identity of the major products was confirmed by sequencing (**Table S7**). Ntds: nucleotide size of sizing marker.

Some [32], [63], but probably not all [29] miRNAs are released as a consequence of MVB's function in loading miRNAs to their target complementary mRNA by the RISC complex. Therefore, we tested if immature miRNAs, which are not expected to associate with RISC are also selectively released into the extracellular space. We assessed several pre-miRNA species, including precursors of preferentially released mature miRNAs (miR-1246, miR-1275, mir-451, miR-638), selectively retained miRNAs (let-7c, let-7f, let-7g, miR-100, miR-23a, miR-27, miR-30c)), and precursors of miRNAs with biological and diagnostic value in breast cancer (miR-155, miR-16, miR-21, miR-200c, miR-221, miR-222) [64], [65]. We quantified the released to retained ratio using primers described in the section Materials and Methods and in **Table S6**. Interestingly, precursors of mature miRNAs that are preferentially released into the extracellular space, including mir-451, miR-1275, miR-1246 were present only in the cellular compartment (**Figure 5A**), confirming that the measured extracellular miRNA is of the mature form only. However, precursors of mature miRNAs that were nearly exclusively detected in the cell, such as let-7c and miR-100, were also detected only in the cells. Furthermore, immature oncomiR miR-155, of which we could not detect the mature form (**Figure 2A**, and data not shown) was also sequestered in cells (**Figure 5A**). All other miRNAs were detected both in the cellular as well as in the extracellular compartment (**Figure 5B**). Furthermore, we detected no correlation between miRNA clusters and the extent of release of their corresponding mature miRNAs (**Figure S2**), suggesting that the release of mature and immature miRNAs is regulated separately.

Quantitation of some miRNAs indicated that in every case tested, more pre-miRNA molecules were retained than released (**Figure 5C**). Recently two pre-miRNAs were also shown to be released from human mesenchymal stem cells [29], suggesting that the release of pre-miRNAs is not limited to MCF7 cells. In an attempt to explain differences in release rates of immature miRNAs, we sequenced some pre-miRNAs to test for modifications including RNA editing [66], yet found none (**Table S7**). Therefore, selection mechanisms also exist for the release of pre-miRNAs, however the nature of this process remains to be established.

A caveat of the quantitation approach used in these studies, is that it does not distinguish primary-miRNA transcripts (pri-miRNAs) from pre-miRNAs [39]. To clarify, we probed for the presence of sequences 5′ of the Drosha processing sites [67] (**Figure 5D**). We focused on 6 miRNAs, miR-200c, let-7c, miR-221, miR-21, miR-23, miR-27 that were released to different extents (**Figure 2D**). We found that the transcripts of 5 of these 6 miRNAs did not extend 5′ of the Drosha-cut site (**Figure 5D**, and data not shown), confirming that we measured *bona fide* pre-miRNAs for these species, and that these miRNAs are released as pre- but not as pri-miRNAs.

However, the transcript containing miR-200c extended 5′ of the stem-loop, as determined by PCR and sequencing (**Figure 5D** **and Table S7**). We tested for pri-miRNA editing, which may explain lack of processing [68], [69] or release, yet found none in the cellular or extracellular population (**Table S7**). These findings suggest that in addition to releasing mature and pre-miRNAs, cells also released miRNA transcripts that were not properly processed either by Drosha or Dicer, including a miRNA locus relevant to breast- and other cancers [40], [70]. The finding of both pre-miRNAs and longer transcripts supports the notion that miRNA transcripts are released in ways in addition to, and independent of those described as a consequence of RISC loading.

### Breast cancer cells release most of their miR-451 and miR-1246 molecules

Some miRNA species were 4 to 34-fold enriched in the extracellular population (**Figure 2**). Interestingly, the absolute concentration of several such species was greater in the extracellular than the intracellular space (**Figures 2A, 2B** **and** **3A**). We considered whether this enriched population was merely overrepresented in the environment, because they constituted the most abundant cellular miRNA component. However, none of the most abundant cellular miRNAs clustered into this category (**Figure 2B**, lower panel). Therefore, properties other than cellular miRNA abundance were responsible for the extent of release of this miRNA subpopulation.

One of the most disproportionately released miRNAs was miR-451, of which more than 90% of the total mature microRNA population was exported into the extracellular space (**Figures 2** **,** **3** **,** **4**). MiR-451 is interesting, because it has been associated with development, maintenance [71], [72], and polarity [73] of epithelial cells. In cancer, miR-451 has been reported to down-regulate macrophage migration inhibitory factor, MIF [74], and multi-drug resistance 1, MDR1 [75], and consequently rendering MCF7 cells more sensitive to the chemotherapeutic agent doxorubicin [76].

We tested if preferential release of miR-1246 and miR-451 was common in breast cancer cell lines, using miR-16 as an internal control. We found that miR-451 and miR-1246 were selectively released from four breast cancer cell lines tested, whereas the release of these miRNAs from nontumorigenic MCF10A cells and unrelated normal fibroblast cell line IMR90 was much lower (**Figure 4**). In addition, the release rate of miR-451 was uniform for MCF7 cells (**Figure 3C**), and accumulation of miR-451 was relatively constant among the four cancer cell lines tested (**Figure 4A–4C**). These data indicate that the release of the majority of miR-451 into the medium is cell-type specific, and perhaps more common in breast cancer cells. These results fit with other studies in which miR-451 was barely detected in MCF7 cells [76], [77]. More importantly, the excessive release of miR-451 might also provide a mechanism to explain how miR-451 accumulates interstitially in breast cancer tumors [77]. Most notably, these data suggest that cells produce some miRNA molecules of which more are released into the environment than are retained.

**Figure 6:**
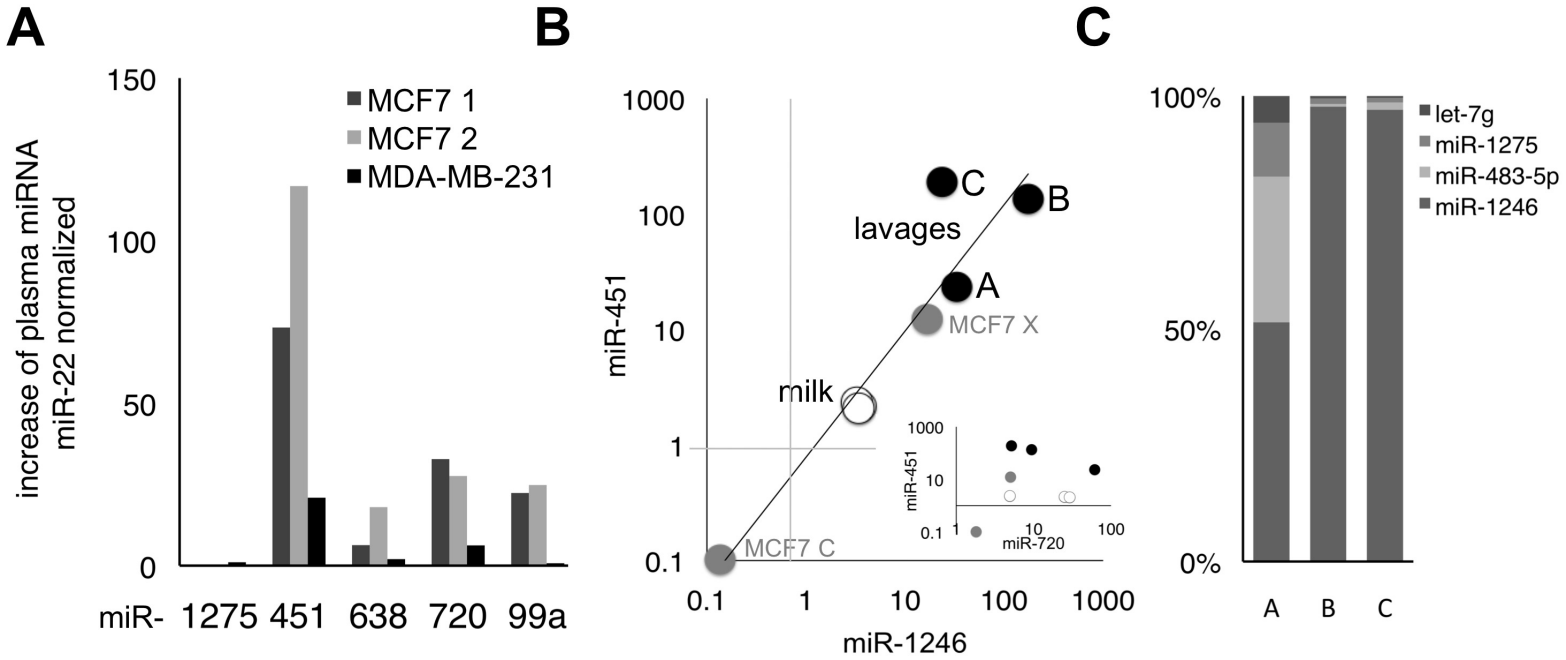
Extracellular Mammary Epithelial Signature miRNAs are Present in Body Fluids. **A**. Abundance of indicated miRNAs quantitated using qRT-PCR from the plasma of mice injected subcutaneously with indicated breast-cancer cell lines as shown in **Figure S3**, and normalized to miR-22, a microRNA of murine blood, but not found in the conditioned media of these cancer cell lines. **B**. Plot of quantities for indicated miRNAs in 3 samples each of human milk (milk), cell-free ductal lavages of breast cancer patients (lavages), extracellular (MCF7 X) and intracellular MCF7 (MCF7 C) preparations, as quantitated by the stem-loop-primer qRT-PCR approach. A: lavages of a patient with atypical ductal hyperplasia; B and C: lavages of patients with less severe epithelial hyperplasia. Inset: plot of ratios for miR-451 and miR-720. Dot shadings correspond to the same samples as labeled in Figure 6B. **C**. Additional miRNAs quantified in ductal lavages using the linker-ligation qRT-PCR method.

We assessed if xenografted MCF7 and MDA-MB-231 cells also release miRNAs into the murine blood circulation (**Figure S3**). To do so, we normalized the measured miRNA levels to miR-22, a miRNA that we did not find to be released from breast cancer cells, but which was present in mouse blood. We found that xenografting either cell line resulted in an increase in plasma levels of miR-451, miR-720, miR-99a (**Figure 6A**). In addition, plasma of mice injected with MCF7 cells, but not plasma of uninjected littermates had detectable amounts of miR-1246 (**Figure S3**). Therefore we conclude that breast cancer cell lines release signature miR-451 and miR-1246 into the blood.

### Ductal fluids contain extracellular miRNA signatures of mammary epithelia

To test whether mammary epithelial cells also release the identified signature miRNAs miR-451, miR-1246, miR-720, and miR-16 in the human body, we focused on fluids that are heavily conditioned by these cells; human milk, which has been reported to contain exosomes [78] and miRNAs [79], and ductal lavages. We found that lavages contained several miRNAs that we found to be abundantly released from HMECs into culture media (**Figure 6B**). Of these, the concentrations of miR-1246, miR-451 and miR-720, normalized to miR-16 in the tested lavages and milk were highly reminiscent of the extracellular miRNA signature, but dissimilar to the intracellular HMEC miRNA signatures (**Figures 6B**). Therefore, we conclude that both mammary fluids contain extracellular miRNA species originating from mammary epithelial cells in a specific conserved ratio.

We also detected differences in the miRNA signatures between milk and lavages. For example, we detected a linear, constant ratio of miR-16-normalized miR-451 to miR-1246 in both milk and lavages, suggesting coordinated release of these two miRNAs. However, the ratios of miR-451 and miR-1246 to miR-16 was much higher in the lavages than the milk, suggesting an overall greater release of these two miRNAs in the lavages compared to the milk (**Figure 6B**). Perhaps, this difference reflects fluid origin. However, the ductal lavages originate from donors that had epithelial atypia (lavages B and C), and atypical ductal hyperplasia (ADH) on biopsy (lavage A, [80]), whereas the milk was from normal donors. This difference raises the possibility that increased miR-451 and miR-1246 concentrations are indicative of the presence of abnormal cells in the mammary gland. In addition, the lavages of the patient with ADH differed in the relative abundance of other miRNAs when compared to the lavages with less severe epithelial atypia (**Figure 6C**), perhaps indicating that different cellular changes induce different modifications in the miRNA composition of ductal lavages.

## Discussion

We report here that cells preferentially release and retain miRNA subpopulations. These results are important when considering circulating miRNAs for diagnosis, and in assessing the biological significance of released miRNAs. For example, the finding that transformed and malignant mammary epithelial cells release most of their miR-451 into the environment has several implications. Interestingly, miR-451 abundance has been reported to correlate with breast cancer in tissue sections. However, miR-451 was low in mammary epithelia, but enriched interstitially [77]. Therefore, miR-451 abundance was attributed to alternate origins, and hence the usefulness of miR-451 in diagnosis was rejected. Yet, miR-451 may have roles in breast- and other cancers [46], [74], [81], and maps to an amplicon that includes HER2 and BRCA1, which are commonly amplified in breast- and other cancers [82], [83], [84]. Therefore, our finding that miR-451 is selectively released from malignant mammary epithelial cells in culture and in the body may be of importance for diagnostics.

In addition, we and others have described that viral RNAs and proteins transferred in exosomes have biological functions relevant to cancer ([23] and reviewed in [26], [85], [86]). In addition vesicular miRNAs have been found to have signaling capacity [27], [30]. Therefore, the finding that some miRNAs are released more abundantly than retained may suggest a role of these miRNAs in signaling. Perhaps selectively released miRNAs, including miR-451 may be involved in paracrine signaling of the cancer and the stroma [87], and in field cancerisation [88].

Alternatively, the release of miRNAs in exosomes might fit with a trash disposal mechanism [89], in which cells release damaged and other cellular components into the environment akin to a house-cleaning mechanism. In agreement with this idea, we have found the accumulation of specific rRNA and snRNA fragments, and improperly processed miRNAs in the extracellular space. However, because specific tRNA fragments have been found to have distinct biological roles [90], RNA fragments may possibly be more than degradation products destined for disposal.

The mechanism of selective miRNA release remains to be determined. For example, in addition to an MVB-mediated miRNAs release mechanism, cell death may be a mechanism of miRNA release [17], [33]. We considered this possibility, and found that IMR90 cells and MDA-MB-231 cells in culture had similar proportions of dead cells (11% and 9% respectively), yet MDA-MB-231 cells released about 2.3 times more of their miR-16 molecules than IMR90 cells did (**Figure 3A**, and data not shown). Therefore, cell death alone cannot explain the accumulation of extracellular miRNAs. To identify the selection mechanism, it may be informative to determine if the selective release and retention of miRNA subpopulations is regulated by environmental cues. In addition, the recent findings that miR-451 biogenesis is dicer-independent [91], raise the possibility that miRNAs processed in noncanonical ways may be specifically targeted for release through mechanisms yet to be determined.

Our finding that the extracellular miRNA profile of ductal fluids and HMECs is similar has several consequences. For example, it opens the possibility of using the extracellular miRNA population of ductal lavages for diagnosis, as is considered for proteins ([92] and reviewed in [93], [94]). This may be especially useful for women at high-risk of breast cancer, where measurements of the extracellular miRNA composition might supplement other approaches, and overcome some of the diagnostic limitations of analyzing the cellular composition of lavages [95], [96]. In addition, the finding of differences in the extracellular miRNA composition of ductal fluids raises an interesting possibility that specific miRNAs contribute to the multifunctional roles milk may have during nursing [79] or consuming animal milk [97], and the function ductal fluid miRNAs may have in the resting gland [98].

## Materials and Methods

### Ethics Statement

This study was conducted according to the principles expressed in the Declaration of Helsinki. The samples were collected under NH/NCI Clinical Protocol # 02-C-0077. The study was approved by the Institutional Review Board of RFUMS (IRB protocol numbers # 001 PATH, # 002 PATH, and # 003 PATH). All patients provided written informed consent for the collection of samples and subsequent analysis.

All animals were handled in strict accordance with good animal practices as defined by the RFUMS IACUC and accredited by the Association for Assessment and Accreditation of Lab Animal Care International (AAALAC) in compliance with the US Public Health Service policy as assured by the Office of Laboratory Animal Welfare. All animal work was approved by the RFUMS IACUC under protocol 08-25.

### Cell Culture

MDA-MB-231, MCF7, MCF 10A, BT-20, IMR90, SK-BR-3 cell lines were purchased from ATCC and maintained according to provider's recommendations, except for MCF 10A, and SK-BR-3 cells. MCF 10A cells were maintained in media containing horse serum as previously described [99]. However, for dot-blot analyses, MCF 10As were grown in calf serum (CS), because horse serum produced a high background reactivity with the secondary antibody. For miRNA analyses, as a rule, MCF10A cells were grown in FBS (Mediatech, Manassas, VA; Gemini, West Sacramento, CA, or HyClone, Thermo Scientific, Pittsburgh, PA), because CS (Gemini, West Sacramento, CA) contained some of the miRNAs measured (**Figure S4A**). Microvesicles including exosomes were depleted from animal sera by filtration and ultracentrifugation approaches as described [23]. Alternatively, most miRNAs tested, including miR-451 were nearly undetectable in the FBS serum we used, consistent with the fact that miR-451 has not been detected in blood microvesicles [34] (**Figure S4**). SK-BR-3 cells were grown in MEM supplemented with 10% FBS, NEAA and sodium pyruvate.

Cells were grown in one 10 cm plate for routine miRNA analyses and 10 15 cm plates for array analyses (2–50*10^6^ cells) for collection of conditioned supernatant and cell pellet. Depending on the cell line used, media was replaced when cells were about 40–70% confluent, and maintained in this medium for 5 days to collect the released particles and miRNAs, unless otherwise indicated. Culturing for 5 days was used to effect optimal exosome accumulation in the media [23]. On the fifth day cells were nearly 90% confluent. Cell viability was quantified using trypan blue as described [100].

### Mice

Nude mice (Taconic, Hudson, NY) were housed, maintained, and injected with indicated breast cancer cell lines according to RFUMS IACUC guidelines, and as described [101]. In brief, 5 million cells of indicated cell lines were injected in 100 µl BD Matrigel (BD Biosciences, San Jose, California) into the right flank of mice, and monitored for tumor growth. Mandibular blood was drawn about weekly according to IACUC protocol and plasma was prepared immediately or after refrigeration.

### Lavages

Coded human breast ductal lavages were collected and provided by Dr. David Danforth (NIH) in accordance to NIH/NCI Institutional Review Board (IRB) guidelines, and were described in [80].

### Milk

Milk was donated from the Mother's Milk Bank of Iowa (The University of Iowa Children's Hospital) and the Indiana Mother's Milk Bank, Inc. Eight hundred microliters of milk from three mothers each was analyzed to determine miRNA composition. The study was approved by the IRB of RFUMS (IRB protocol number # 001 PATH).

### Exosome Preparation

P70 and S70 was prepared as described [23]. In brief, conditioned media or sera were cleared of cells and cell debris by low-speed centrifugation and filtration, followed by concentrating the remaining particulate at 70,000 g for 1 h. The pellet was washed in PBS by resuspension, and centrifuged again at 70,000 g. We chose this centrifugation speed over conventional higher speed-preparations [102], because vesicles prepared at higher speeds can lose some of their biological activities [23].

### Negative-Stain EM

EM was done as described [23]. In brief, a P70 was fixed in suspension with 2% PFA and 0.2% glutaraldehyde followed by pelleting. This preparation was adsorbed to butvar-coated grids followed by negative staining/embedding in 1% aqueous uranyl acetate in 1% methyl cellulose [103]. Micrographs were acquired using a transmission electron microscope (H7000T; Hitachi).

### Dot-Blots and Slot-Blots

Blots were prepared using antibodies as described [23]. In brief, P70s and other preparations were resuspended in Tris-buffered saline and blotted onto Immobilon FL (Millipore) or nitrocellulose (Whatman) using a Bio-Dot Filtration Apparatus (BioRad). Antibody binding was quantified using a Typhoon 9400 (GE Healthcare), and goat-anti-mouse IgG-Alexa 488 (Invitrogen) and ImageQuant T software. Antigenicity was determined by quantifying antibodies bound to slot-or dot blotted P70 and other material using a standard curve produced using dilutions of a known amount of primary antibody.

### RNA Extraction

Total RNA was extracted using Trizol procedure (Invitrogen). For linker ligation qRT-PCR, small RNAs were enriched from Trizol-extracted total RNA using PureLink (Invitrogen) according to manufacturer's instructions. For looped-primer qRT-PCR, total RNA was extracted using Trizol.

### 5′ End Labeling of RNA

RNA (∼50 pmol) was isolated from MCF7 cells or exosomes and enriched for small RNAs using PureLink followed by treatment with with Antarctic phosphatase (NEB) according to manufacturers protocol. Dephosphorylated RNA was 5′ end labeled using T4 polynucleotide kinase (-3′ phosphatase minus) (NEB) and ^32^P-γ-ATP according to the manufacturers instructions. Radiolabeled RNA was separated on a 12% urea-polyacrylamide gel.

### miRNA Microarrays

MicroRNAs were screened by LC Sciences. One microgram of MCF7 cellular RNA preparation was labeled with Cy5, and one microgram of the extracellular RNA samples was labeled with Cy3. In brief, the RNA was labeled with Cy5 or Cy3 and hybridized to LCSciences standard arrays for mature miRNA of all species available in the Sanger miRBase database (Release 12.0). The data were analyzed including background subtraction, using a LOWESS (locally weighted regression) method on the background-subtracted data. Only transcripts with a signal intensity higher than 3× (background SD) and spot CV<0.5. CV was calculated by (SD)/(signal intensity), and in which repeating probes on the array produced signals from at least 50% of the repeating probes are above detection level. Only data are plotted in which extracellular or the cellular signal intensity for a particular transcript was at least 100. Array data have been submitted to the NCBI/GEO database, GPL10517 - LC_MRA-1001_miRHuman_12.0_080901, GSE22235.

### miRNA Quantitation

Two PCR approaches were used, stem-loop primers, which in general are more sensitive and specific, and linker-ligated primers, which allow sequencing of the 3′ portion of miRNAs, and the quantitation of a wider variety of miRNAs from the same cDNA sample.

### RNA Ligation

For linker-ligated qRT-PCR, the 3′ end of RNA was ligated to linker 1 (IDT, Coralville, IA) according to manufacturer instructions for miRCat (IDT), except for the substitution of Ligation Enhancer with PVA, and truncated RNA ligase 2 (NEB) to promote polar ligation.

### Reverse-Transcription

Reverse Transcription (RT) was carried out using SuperScript III (Invitrogen) according to manufacturer's instructions, and using half of the total RNA or microRNA isolated from the P70, cells, or other sources and using 20 pmoles of the primers discussed below. For linker ligation, Modban primer [38] was used for reverse transcription. For reverse transcription of mature miRNAs, we used sets of stem-looped primers (**Table S5**). For pre-miRNAs and other immature miRNAs, we used a set of primers specific for the 3′ end of immature miRNAs (**Table S6**), many of which are described in [39].

### Quantitative PCR

qPCR was performed on a 1/1000 dilution of the cDNA using the primers listed in **Tables S5 and S6**. The optimal concentration of template used was determined empirically (**Figure S5**). qPCR was performed in triplicate 20 µl reactions using an ABI 7500 Real-Time PCR System (Applied Biosystems, Carlsbad, CA). The reagents used included PCR primers produced by IDT, Invitrogen or Operon and Power SYBR Green PCR Master Mix. We optimized the qRT-PCR approach empirically to produce a single band of the correct size by native PAGE (**Figure 2E**). In so doing, we were able to confirm the specificity and purity of our amplified and quantitated products. Quantitation was performed for up to 40 cycles of melting at 95°C for 15 s, annealing at 50°C for 1 min, and extension at 73°C for 15 s, the optimal conditions determined empirically. Standard PCR was performed on a MultiGene Thermal Cycler (Labnet International, Inc, Edison, NJ) using PCR with GoTaq polymerase (Promega).

### Absolute Quantification

MiRNA abundance was measured by computing attomoles based on comparing CT values of samples to dilutions of a synthetic cDNA of the same miRNA sequence to make a standard curve.

### Relative Quantification

Relative quantification was performed using the ABI 7500 detection software with correction for amplification efficiency based on an exponential model of PCR [104], [105], and after normalizing miRNA recovery to spiked control RNA (INT-RNA) of known quantity (**Table S4**).

### Quantitation of Pre-miRNA Sequences

Quantitation of pre-miRNA sequences was performed with the primers listed in **Table S6**. To confirm pre-miRNA sequences analyzed, we sequenced the PCR products (**Table S7**). To determine proper processing of the pre-miRNAs, we designed two upstream primers (**US1 and US2**) to the region 5′ of the stem-loops of the pri-miRNAs, and a downstream primer to the 3′ end of the immature miRNAs (Rm) for amplification by PCR. (**Table S6**). The resulting products of expected size were sequenced to confirm their identity (**Table S7**).

### PAGE

Final PCR products were separated on a 12% native acrylamide gel at room temperature, stained with SYBR Gold (Invitrogen), and photographed using Gene Genius Bioimaging System, and GeneSnap software (Syngene, Frederick, MD).

### Cloning and Sequencing

PCR products were cloned into pGEM-T Easy (Promega, Madison, WI) and sequenced at the DNA sequencing facility of the University of Chicago Cancer Research Center. By sequencing the stem-loop and linker-ligation products, we determined that miR-100 identified by the array (**Figure 2A**) was in fact miR-99a, which differs from miR-100 by a single nucleotide (**Table S5**).

## Supporting Information

Figure S1 Some Diagnostic miRNAs are Mostly Retained. Indicated miRNAs were amplified from RNA collected from cells and the conditioned media of MCF7 cells using looped primers, and separated by native PAGE. A miRNAs in which the cellular (c), but not the extracellular (x) RNA population contained the indicated miRNAs. In each case of the cellular sample, the main band (arrow) was excised, cloned and sequenced and found to be the expected amplified product. In the case of extracellular samples the main bands, all of which migrated differently (star), had no resemblance to the miRNA to be amplified as determined by sequencing. B miRNAs in which the released miRNA population contained a band of much lower abundance than the retained population.
(0.31 MB PDF)

Figure S2 Lack of Correlation between Primary Transcript and Release Rate of MiRNAs. Released/retained miRNAs (x/c) as evaluated in Figure 1 are plotted according to chromosomal location (chromosomes 1-X) from top to bottom. Micro-RNA clusters are indicated in color. Note that for cluster miR-200c-141, which is located in a single intron, 30 times more of its encoded miR-200c molecules than miR-141 molecules were released from cells (Figure 2A) than retained, indicating that the extent of miRNA release is not determined at the primary-miRNA level. In support of this idea, within the other miR-200 cluster, which encodes miR-200a, miR-200b and miR-429 (Bracken CP, Gregory PA, Kolesnikoff N, Bert AG, Wang J, Shannon MF, Goodall GJ. A doublenegative feedback loop between ZEB1-SIP1 and the microRNA-200 family regulates epithelialmesenchymal transition. Cancer Res. 2008 Oct 1;68(19):7846–54), more than half of miR-429 and miR-200b molecules are released, whereas most of miR-200a is retained. Therefore, the extracellular accumulation of mature miRNAs is regulated at levels other than the primary transcript abundance. MicroRNAs contained in particular clusters are indicated by red or green colored bars.
(2.32 MB PDF)

Figure S3 MiR-1246 is a Reliable Indicator of Body Fluids Conditioned by Mammary Epithelia. A tumor growth of xenografted cells of mice used for assessment of miRNA abundance in blood plasma as indicated in Figure 4A. The tumor size is presented as the product of the 3 diameters. Data presented in Figure 6 are from bleeds at day 30 (arrow). B PAGE of end-point PCR of miR-1246 of bleeds of MCF7-1 and MDA-MB-231.
(0.20 MB PDF)

Figure S4 Fetal Bovine Serum Does Not Interfere With Extracellular MiRNA Assessment. A Relative abundance of indicated miRNAs in calf serum (CS) and fetal bovine serum (FBS), normalized to INT-RNA. Note the absence of miR-451 from FBS, but that CS contains measurable levels of Bos taurus miR-451, which differs from hsa-miR-451 by a single terminal nucleotide (Long JE, Chen HX. Identification and characteristics of cattle microRNAs by homology searching and small RNA cloning. Biochem Genet. 2009 Jun;47(5–6):329–43). B End-point PCR using stem-loop primers on extracellular (x) and cellular (c) miR-451 of indicated breast cancer cell lines grown in complete FBS, or in FBS depleted of microvesicles (FBS S100). C Ratio of miRNAs in c and x of cells grown in complete FBS and FBS depleted of microvesicles (FBS S100). D. MiRNAs measured as in Supplemental Figure S4C, by qRT-PCR using linker-ligation. Error bars indicate standard deviation. A is an average of 2 experiments, C and E are averages of 3 independent experiments.
(1.28 MB PDF)

Figure S5 Template-independent amplification products at low template concentrations. Mature miR-16 was assessed by the stem-loop-primer protocol on 5 fold serial dilutions of a synthetic DNA construct reflecting the expected product of miR-16.
(0.22 MB PDF)

Table S1 RRNA and snRNA fragments detected in unique RNA band of extracellular miRNAs. RNA sequences cloned and sequenced from bands in Figure 1, D and E, marked with a star.
(0.03 MB DOC)

Table S2 RNA Subpopulation Enriched in the Extracellular Space. Sequences identified with D1, D2, D3 represent sequence data of miRNAs extracted from cells, all others are miRNA sequences retrieved from the extracellular space.
(0.05 MB DOC)

Table S3 Oligonucleotides used for Mature miRNA Quantitation.
(0.07 MB DOC)

Table S4 Stem Looped Primers used for Simultaneous Analyses of Multiple Transcripts.
(0.05 MB DOC)

Table S5 Confirmed sequences of qPCR products quantified using linker ligated primers.
(0.03 MB DOC)

Table S6 Primers for Quantifying pre-miRNAs and Other Immature miRNAs.
(0.07 MB DOC)

Table S7 Sequences of Immature miRNAs Detected.
(0.05 MB DOC)
