# Supplementary figures and images for: Selective Release of MicroRNA Species from Normal and Malignant Mammary Epithelial Cells
Source: PLoS One. 2010 Oct 20;5(10):e13515. doi: 10.1371/journal.pone.0013515 (PMC2958125; doi:10.1371/journal.pone.0013515)

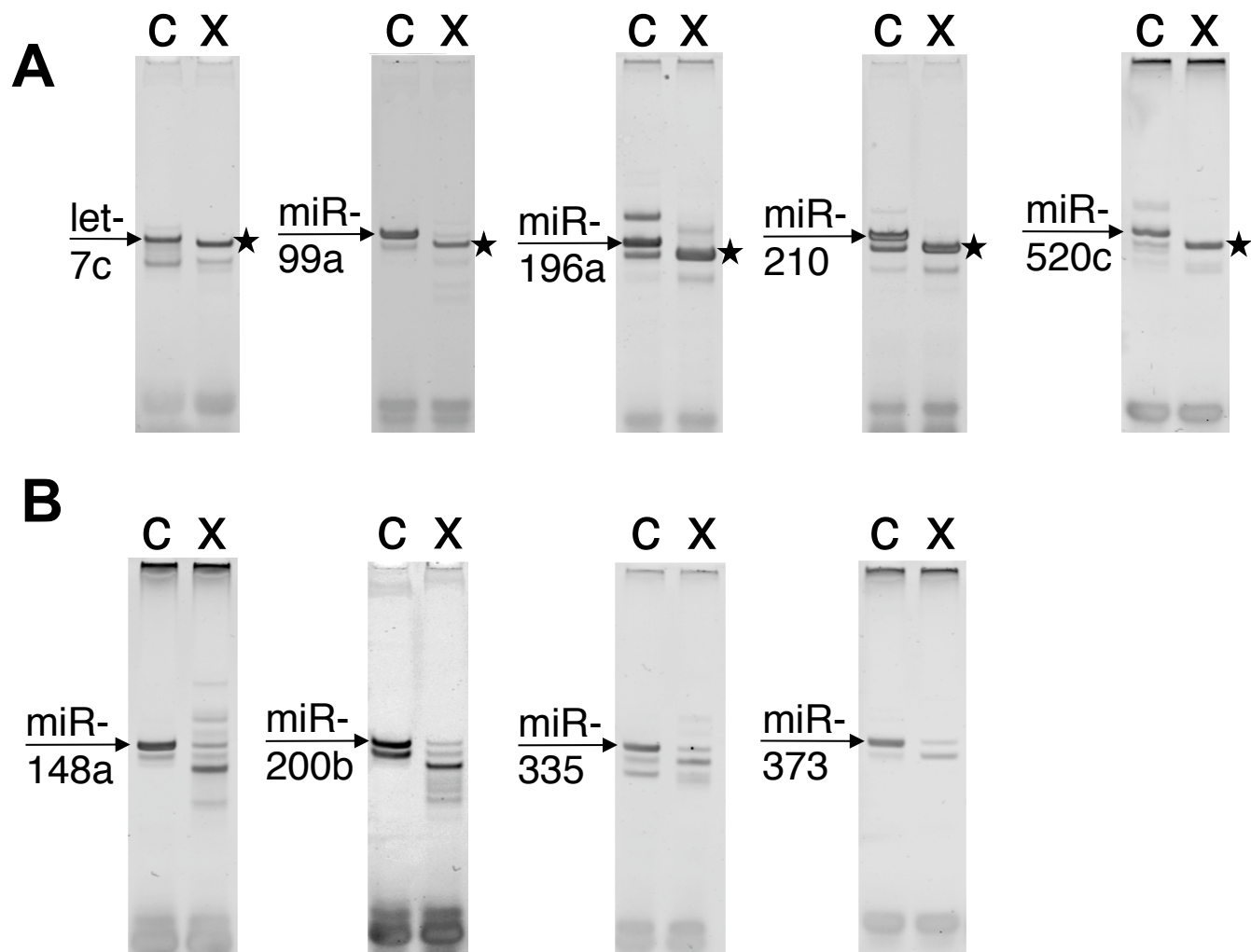

Supplement: Figure S1 — Some Diagnostic miRNAs are Mostly Retained. Indicated miRNAs were amplified from RNA collected from cells and the conditioned media of MCF7 cells using looped primers, and separated by native PAGE. A miRNAs in which the cellular (c), but not the extracellular (x) RNA population contained the indicated miRNAs. In each case of the cellular sample, the main band (arrow) was excised, cloned and sequenced and found to be the expected amplified product. In the case of extracellular samples the main bands, all of which migrated differently (star), had no resemblance to the miRNA to be amplified as determined by sequencing. B miRNAs in which the released miRNA population contained a band of much lower abundance than the retained population. (0.31 MB PDF) [file pone.0013515.s001.pdf]

**A**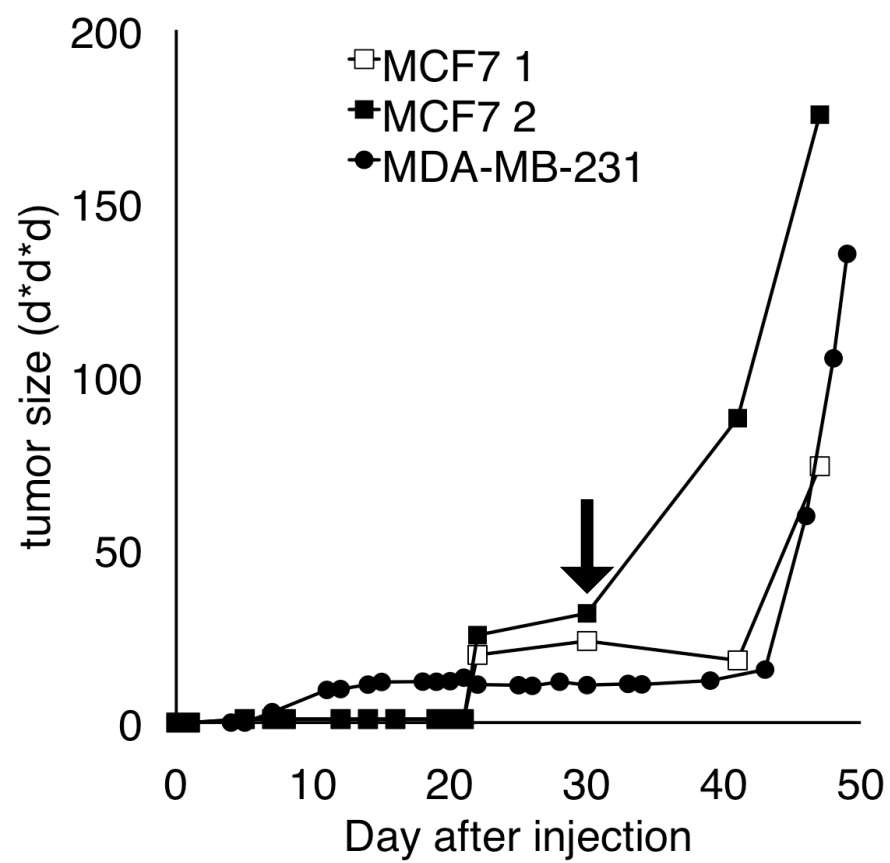**B**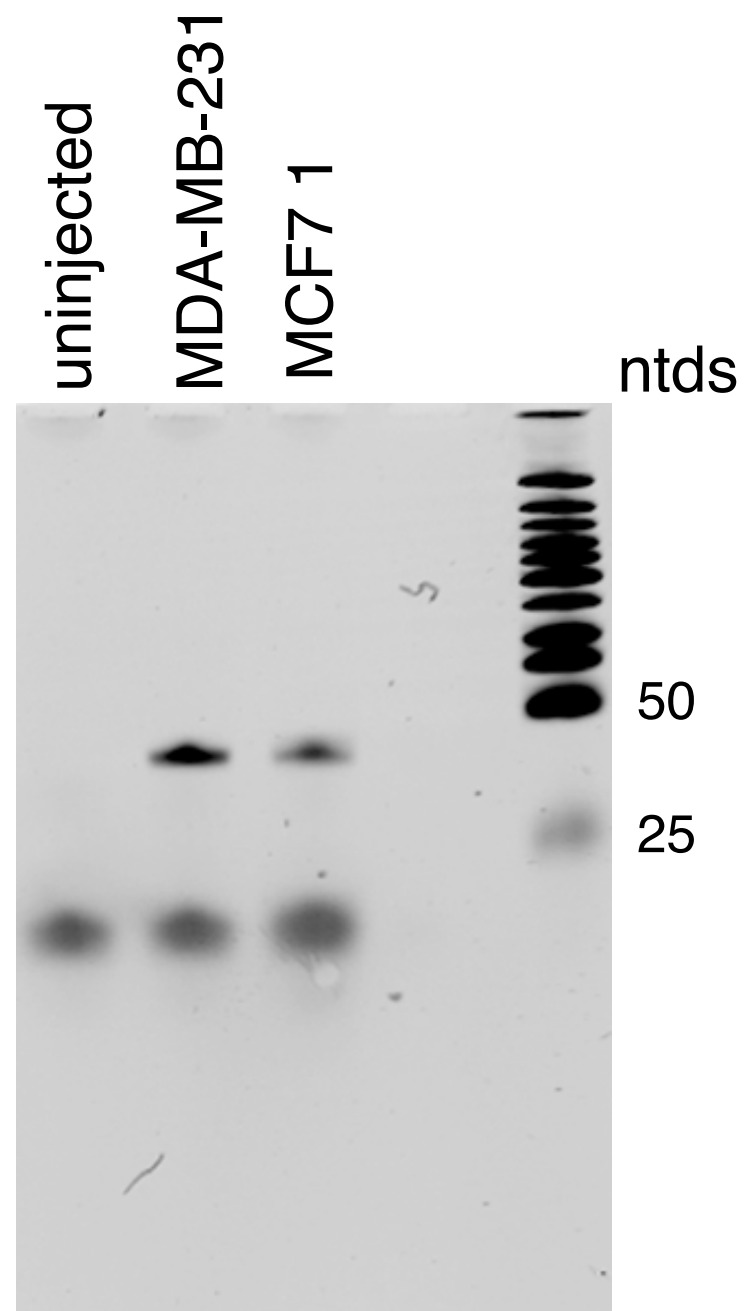

Supplement: Figure S3 — MiR-1246 is a Reliable Indicator of Body Fluids Conditioned by Mammary Epithelia. A tumor growth of xenografted cells of mice used for assessment of miRNA abundance in blood plasma as indicated in Figure 4A. The tumor size is presented as the product of the 3 diameters. Data presented in Figure 6 are from bleeds at day 30 (arrow). B PAGE of end-point PCR of miR-1246 of bleeds of MCF7-1 and MDA-MB-231. (0.20 MB PDF) [file pone.0013515.s003.pdf]

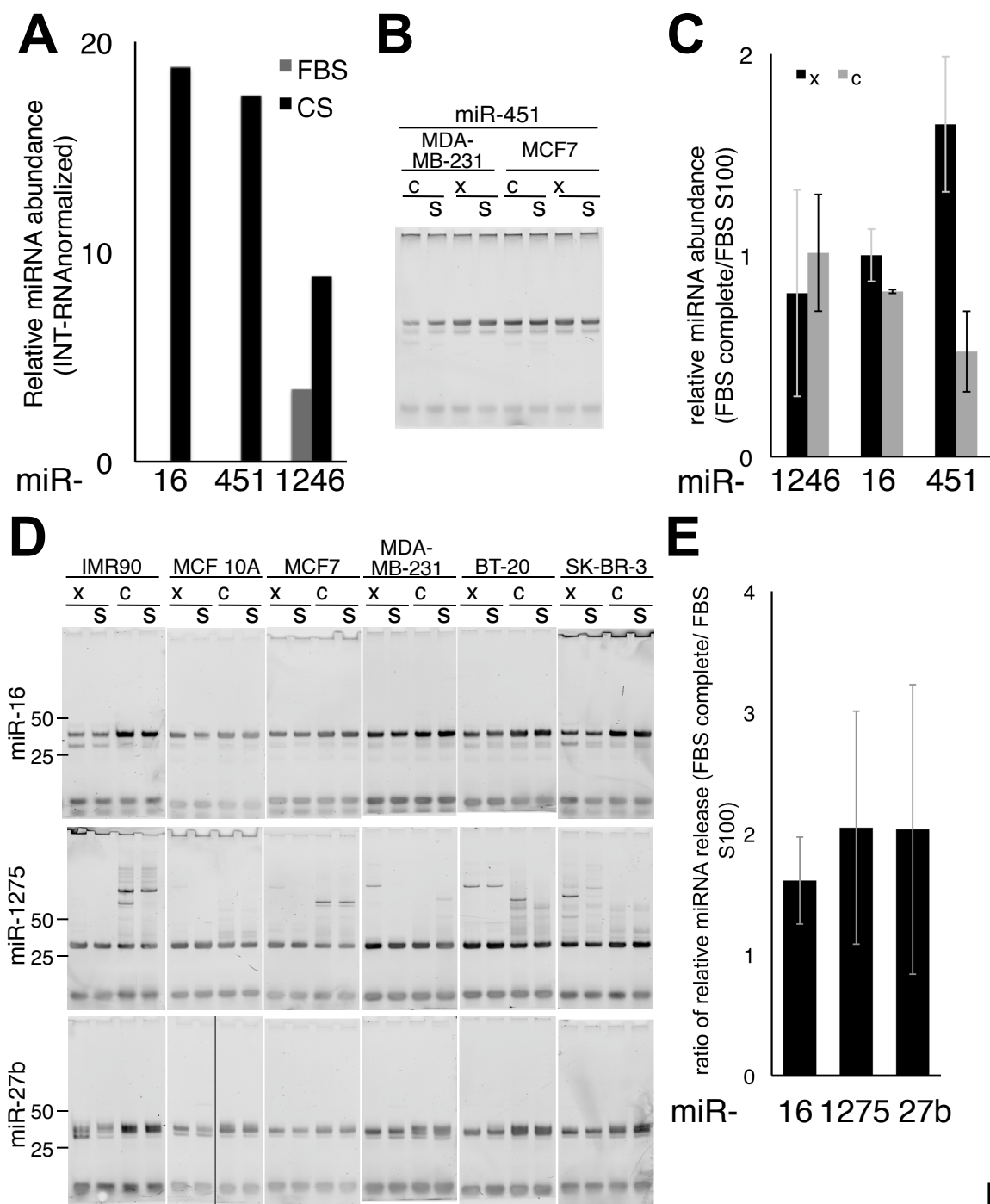

Supplement: Figure S4 — Fetal Bovine Serum Does Not Interfere With Extracellular MiRNA Assessment. A Relative abundance of indicated miRNAs in calf serum (CS) and fetal bovine serum (FBS), normalized to INT-RNA. Note the absence of miR-451 from FBS, but that CS contains measurable levels of Bos taurus miR-451, which differs from hsa-miR-451 by a single terminal nucleotide (Long JE, Chen HX. Identification and characteristics of cattle microRNAs by homology searching and small RNA cloning. Biochem Genet. 2009 Jun;47(5–6):329–43). B End-point PCR using stem-loop primers on extracellular (x) and cellular (c) miR-451 of indicated breast cancer cell lines grown in complete FBS, or in FBS depleted of microvesicles (FBS S100). C Ratio of miRNAs in c and x of cells grown in complete FBS and FBS depleted of microvesicles (FBS S100). D. MiRNAs measured as in Supplemental Figure S4C, by qRT-PCR using linker-ligation. Error bars indicate standard deviation. A is an average of 2 experiments, C and E are averages of 3 independent experiments. (1.28 MB PDF) [file pone.0013515.s004.pdf]

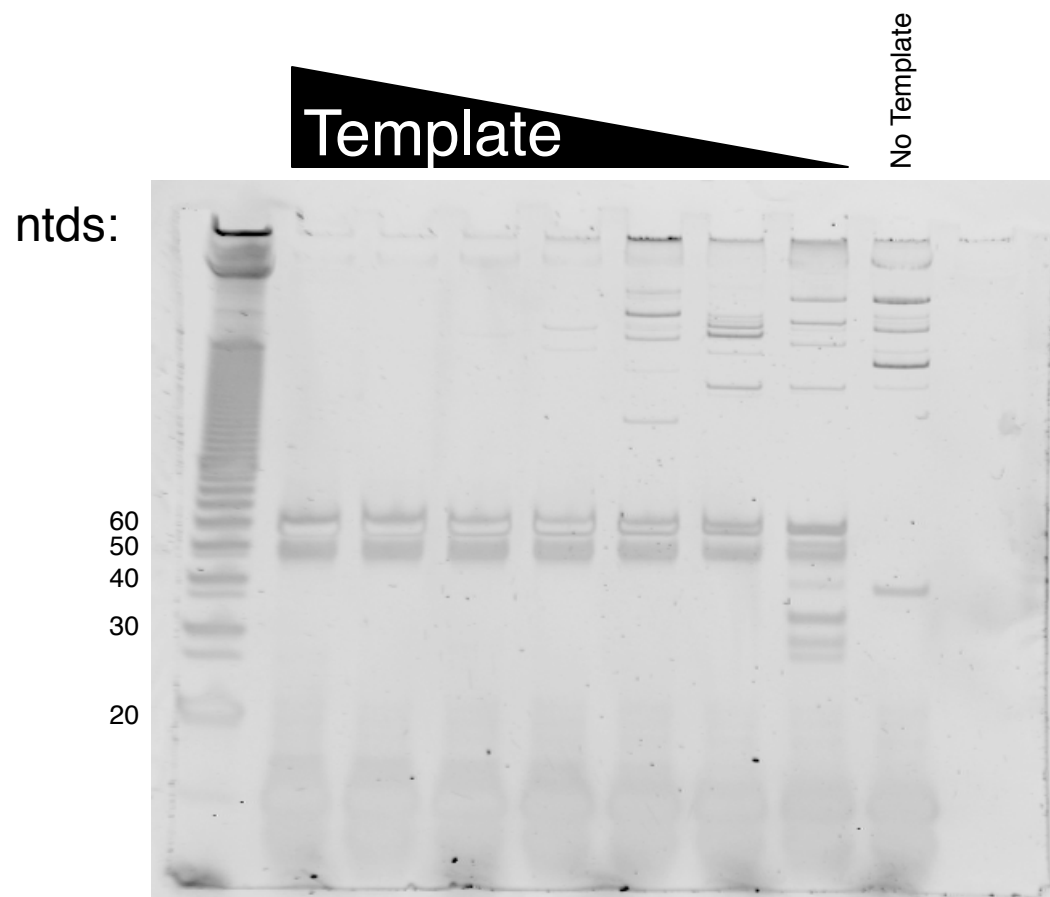

Pigati et al. Supplemental Figure S5

Supplement: Figure S5 — Template-independent amplification products at low template concentrations. Mature miR-16 was assessed by the stem-loop-primer protocol on 5 fold serial dilutions of a synthetic DNA construct reflecting the expected product of miR-16. (0.22 MB PDF) [file pone.0013515.s005.pdf]
