# Supplementary material for: Selective Release of MicroRNA Species from Normal and Malignant Mammary Epithelial Cells
Source: PLoS One. 2010 Oct 20;5(10):e13515. doi: 10.1371/journal.pone.0013515 (PMC2958125; doi:10.1371/journal.pone.0013515)
Supplement: Figure S2 — Lack of Correlation between Primary Transcript and Release Rate of MiRNAs. Released/retained miRNAs (x/c) as evaluated in Figure 1 are plotted according to chromosomal location (chromosomes 1-X) from top to bottom. Micro-RNA clusters are indicated in color. Note that for cluster miR-200c-141, which is located in a single intron, 30 times more of its encoded miR-200c molecules than miR-141 molecules were released from cells (Figure 2A) than retained, indicating that the extent of miRNA release is not determined at the primary-miRNA level. In support of this idea, within the other miR-200 cluster, which encodes miR-200a, miR-200b and miR-429 (Bracken CP, Gregory PA, Kolesnikoff N, Bert AG, Wang J, Shannon MF, Goodall GJ. A doublenegative feedback loop between ZEB1-SIP1 and the microRNA-200 family regulates epithelialmesenchymal transition. Cancer Res. 2008 Oct 1;68(19):7846–54), more than half of miR-429 and miR-200b molecules are released, whereas most of miR-200a is retained. Therefore, the extracellular accumulation of mature miRNAs is regulated at levels other than the primary transcript abundance. MicroRNAs contained in particular clusters are indicated by red or green colored bars. (2.32 MB PDF) [file pone.0013515.s002.pdf]

Supplemental Table S1. RRNA and snRNA fragments detected in unique RNA band of extracellular miRNAs

(Figure 1, D and E, star)

Table S1A

Hs 5.8S ribosomal RNA

```
1           GACTCTTAGC GGTGGATCAC TCGGCTCGTG CGTCGATGAAG AACGCAGCTAG
51          CTGCGAGAAT TAATGTGAAT TGCAGGACAC ATTGATCATCG ACACTTCGAAC
101         GCACTTGCGG CCCCGGGTTC CTCCCGGGGC TACGCCTGTCT GAGCGTCGCTT
2_B1                              CTCCCGGGGC TACGCCTGTCT GAGCGTCGC
1_B2                              CTCCCGGGGC TACGCCTGTCT GAGCGTCGC
1_D2                              CTCCCGGGGC TACGCCTGTCT GAGCGTCGC
2_E2                            C CTCCCGGGGC TACGCCTGTCT GAGCGTCGCT
```

Table S1B

Hs U1A small nuclear RNA (RNU1A)

```
1           ATACTTACCT GGCAGGGGAG ATACCATGAT CACGAAGGTG GTTTTCCCAG
2_B3                           GAG ATACCATGAT CACGAAGGTG GTTTTCCCAG
1_E3                           GAG ATACCATGAT CACGAAGGTG GTTTTCCCAG

51          GGCGAGGCTT ATCCATTGCA CTCCGGATGT GCTGACCCCT GCGATTTCCC
2_B3        GGCGAGGC
1_E3        GGCGAGGC

101         CAAATGTGGG AAACTCGACT GCATAATTTG TGGTAGTGGG GGACTGCGTT
151         CGCGCTTTCC CCTG
```

Supplemental Table S2. RNA Subpopulation Enriched in the Extracellular Space

(D1, D2, D3: cellular sequences, others, extracellular.)

1-5035 Homo sapiens 28S ribosomal RNA (LOC100008589), NR_003287

```
1     CGCGACCTCA GATCAGACGT GGCGACCCGC TGAATTTAAG CATATTAGTC AGCGGAGGAA AAGAAACTAA CCAGGATTCC CTCAGTAACG GCGAGTGAAC
mir-923                                             TATTTGTC AGCGGAGGAA AAGAAACTAA CCAGGATTCC CTCAGTAATG GCGAGTG
G2                                                                                                 TAACG GCGAGTGAAC
D3                                                                                                 TAACG GCGAGTGAAC
C3                                                                                              AGGTAACG GCGAGTGAAC
D1                                                                                                                TGAAC
G1                                                                                                                TGAAC
E1                                                                                                                TAGGT

101   AGGGAAGAGC CCAGCGCCGA ATCCCCGCCC CGCGGGGCGC GGGACATGTG GCGTACGGAA GACCCGCTCC CCGGCGCCGC TCGTGGGGGG CCCAAGTCCT
G2    AGGGAAGAGC CCAGCGCCGA ATCCCCGCCC CGC
D3    AGGGAAGAGC CCAGCGCCGA ATCCCCGCCC CGC
C3    AGGGAAGAGC CCAGCGCCGA ATCCCCGCCC CGC
D1    AGGGAAGAGC CCAGCGCCGA ATCCCCGCCC CGC
G1    AGGGAAGAGC CCAGCGCCGA ATCCCCGCCC CGC
C2    AGGGAAGAGC CCAGCGCCGA ATCCCCGCCC CGC
E1    AGGGAAGAGC CCAGCGCCGA ATCCCCGCCC CGC
B3                                       GGCGGGGCGC GGGACATGTG GCGTACGGAA GACCCGC
D2                                       AGGGGGGCGC GGGACATGTG GCGTACGGAA GACCCGC

201   TCTGATCGAG GCCCAGCCCG TGGACGGTGT GAGGCCGGTA GCGGCCGGCG CGCGCCCGGG TCTTCCCGGA GTCGGGTTGC TTGGGAATGC AGCCCAAAGC
301   GGGTGGTAAA CTCCATCTAA GGCTAAATAC CGGCACGAGA CCGATAGTCA ACAAGTACCG TAAGGGAAAG TTGAAAAGAA CTTTGAAGAG AGAGTTCAAG
401   AGGGCGTGAA ACCGTTAAGA GGTAAACGGG TGGGGTCCGC GCAGTCCGCC CGGAGGATTC AACCCGGCGG CGGGTCCGGC CGTGTCGGCG GCCCGGCGGA
```

501 TCTTTCCCGC CCCCCGTTCC TCCCGACCCC TCCACCCGCC CTCCCTTCCC CCGCCGCCCC TCCTCCTCCT CCCCGGAGGG GGCGGGCTCC GGCGGGTGCG
601 GGGGTGGGCG GGCGGGGCCG GGGGTGGGGT CGGCGGGGGA CCGTCCCCCG ACCGGCGACC GGCCGCCGCC GGGCGCATTT CCACCGCGGC GGTGCGCCGC
701 GACCGGCTCC GGGACGGCTG GGAAGGCCCG GCGGGGAAGG TGGCTCGGGG GGCCCCGTCC GTCCGTCCGT CCTCCTCCTC CCCCGTCTCC GCCCCCCGGC
A2 GACCGGCTCC GGGACGGCTG GGAAGGCCCG GCGGGGAAGG T
C2 GACCGGCTCC GGGACGGCTG GGAAGGCCCG GCGGGGAAGG T
E2 TCCTCCTC CCCCGTCTCC GCCCCCCGGC

801 CCCGCGTCCT CCCTCGGGAG GGCGCGCGGG TCGGGGCGGC GGCGGCGGCG GCGGTGGCGG CGGCGGCGGG GGCGGCGGGA CCGAAACCCC CCCCGAGTGT
E2 CCCGCGTCCT CCCT

901 TACAGCCCCC CCGGCAGCAG CACTCGCCGA ATCCCGGGGC CGAGGGAGCG AGACCCGTCG CCGCGCTCTC CCCCCTCCCG GCGCCCACCC CCGCGGGGAA
G2 CGAGGGAGCG AGACCCGTCG CCGCGCT

1001 TCCCCCGCGA GGGGGGTCTC CCCCGCGGGG GCGCGCCGGC GTCTCCTCGT GGGGGGGCCG GGCCACCCCT CCCACGGCGC GACCGCTCTC CCACCCCTCC
1101 TCCCCGCGCC CCCGCCCCGG CGACGGGGGG GGTGCCGCGC GCGGGTCGGG GGGCGGGGCG GACTGTCCCC AGTGCGCCCC GGGCGGGTCG CGCCGTCGGG
1201 CCCGGGGGAG GTTCTCTCGG GGCCACGCGC GCGTCCCCCG AAGAGGGGGA CGGCGGAGCG AGCGCACGGG GTCGGCGGCG ACGTCGGCTA CCCACCCGAC
1301 CCGTCTTGAA ACACGGACCA AGGAGTCTAA CACGTGCGCG AGTCGGGGGC TCGCACGAAA GCCGCCGTGG CGCAATGAAG GTGAAGGCCG GCGCGCTCGC
1401 CGGCCGAGGT GGGATCCCGA GGCCTCTCCA GTCCGCCGAG GGCGCACCAC CGGCCCGTCT CGCCCGCCGC GCCGGGGAGG TGGAGCACGA GCGCACGTGT
1501 TAGGACCCGA AAGATGGTGA ACTATGCCTG GGCAGGGCGA AGCCAGAGGA AACTCTGGTG GAGGTCCGTA GCGGTCCTGA CGTGCAAATC GGTCGTCCGA
1601 CCTGGGTATA GGGGCGAAAG ACTAATCGAA CCATCTAGTA GCTGGTTCCC TCCGAAGTTT CCCTCAGGAT AGCTGGCGCT CTCGCAGACC CGACGCACCC
1701 CCGCCACGCA GTTTTATCCG GTAAAGCGAA TGATTAGAGG TCTTGGGGCC GAAACGATCT CAACCTATTC TCAAACTTTA AATGGGTAAG AAGCCCGGCT
1801 CGCTGGCGTG GAGCCGGGCG TGGAATGCGA GTGCCTAGTG GGCCACTTTT GGTAAGCAGA ACTGGCGCTG CGGGATGAAC CGAACGCCGG GTTAAGGCGC
1901 CCGATGCCGA CGCTCATCAG ACCCCAGAAA AGGTGTTGGT TGATATAGAC AGCAGGACGG TGGCCATGGA AGTCGGAATC CGCTAAGGAG TGTGTAACAA
2001 CTCACCTGCC GAATCAACTA GCCCTGAAAA TGGATGGCGC TGGAGCGTCG GGCCCATACC CGGCCGTCGC CGGCAGTCGA GAGTGGACGG GAGCGGCGGG
2101 GGCGGCGCGC GCGCGCGCGC GTGTGGTGTG CGTCGGAGGG CGGCGGCGGC GGCGGCGGCG GGGGTGTGGG GTCCTTCCCC CGCCCCCCCC CCCACGCCTC
C3 TG CGTCGGAGGG CGGCGGCGGC GGCGGCGGCG GGGGT
2A TG CGTCGGAGGG CGGCGGCGGC GGCGGCGGCG GGGGT

2201 CTCCCCTCCT CCCGCCCACG CCCCGCTCCC CGCCCCCGGA GCCCCGCGGA CGCTACGCCG CGACGAGTAG GAGGGCCGCT GCGGTGAGCC TTGAAGCCTA
2301 GGGCGCGGGC CCGGGTGGAG CCGCCGCAGG TGCAGATCTT GGTGGTAGTA GCAAATATTC AAACGAGAAC TTTGAAGGCC GAAGTGGAGA AGGGTTCCAT
2401 GTGAACAGCA GTTGAACATG GGTCAGTCGG TCCTGAGAGA TGGGCGAGCG CCGTTCCGAA GGGACGGGCG ATGGCCTCCG TTGCCCTCGG CCGATCGAAA
2501 GGGAGTCGGG TTCAGATCCC CGAATCCGGA GTGGCGGAGA TGGGCGCCGC GAGGCGTCCA GTGCGGTAAC GCGACCGATC CCGGAGAAGC CGGCGGGAGC
2601 CCCGGGGAGA GTTCTCTTTT CTTTGTGAAG GGCAGGGCGC CCTGGAATGG GTTCGCCCCG AGAGAGGGGC CCGTGCCTTG GAAAGCGTCG CGGTTCCGGC
G3 GT CCTGGAATGG GTTC

2701 GGCGTCCGGT GAGCTCTCGC TGGCCCTTGA AAATCCGGGG GAGAGGGTGT AAATCTCGCG CCGGGCCGTA CCCATATCCG CAGCAGGTCT CCAAGGTGAA
2801 CAGCCTCTGG CATGTTGGAA CAATGTAGGT AAGGGAAGTC GGCAAGCCGG ATCCGTAACT TCGGGATAAG GATTGGCTCT AAGGGCTGGG TCGGTCGGGC
2901 TGGGGCGCGA AGCGGGGCTG GGCGCGCGCC GCGGCTGGAC GAGGCGCGCG CCCCCCCCAC GCCCGGGGCA CCCCCCTCGC GGCCCTCCCC CGCCCCACCC
3001 GCGCGCGCCG CTCGCTCCCT CCCCACCCCG CGCCCTCTCT CTCTCTCTCT CCCCCGCTCC CCGTCCTCCC CCCTCCCCGG GGGAGCGCCG CGTGGGGGCG
3101 CGGCGGGGGG AGAAGGGTCG GGGCGGCAGG GGCCGCGCGG CGGCCGCCGG GGCGGCCGGC GGGGGCAGGT CCCCGCGAGG GGGGCCCCGG GGACCCGGGG
3201 GGCCGGCGGC GGCGCGGACT CTGGACGCGA GCCGGGCCCT TCCCGTGGAT CGCCCCAGCT GCGGCGGGCG TCGCGGCCGC CCCCGGGGAG CCCGGCGGCG
3301 GCGCGGCGCG CCCCCCACCC CCACCCCACG TCTCGGTCGC GCGCGCGTCC GCTGGGGGCG GGAGCGGTCG GGCGGCGGCG GTCGGCGGGC GGCGGGGCGG
3401 GGCGGTTCGT CCCCCCGCCC TACCCCCCCG GCCCCGTCCG CCCCCCGTTC CCCCCTCCTC CTCGGCGCGC GGCGGCGGCG GCGGCAGGCG GCGGAGGGGC
3501 CGCGGGCCGG TCCCCCCCGC CGGGTCCGCC CCCGGGGCCG CGGTTCCGCG CGCGCCTCGC CTCGGCCGGC GCCTAGCAGC CGACTTAGAA CTGGTGCGGA
3601 CCAGGGGAAT CCGACTGTTT AATTAAAACA AAGCATCGCG AAGGCCCGCG GCGGGTGTTG ACGCGATGTG ATTTCTGCCC AGTGCTCTGA ATGTCAAAGT
3701 GAAGAAATTC AATGAAGCGC GGGTAAACGG CGGGAGTAAC TATGACTCTC TTAAGGTAGC CAAATGCCTC GTCATCTAAT TAGTGACGCG CATGAATGGA
3801 TGAACGAGAT TCCCACTGTC CCTACCTACT ATCCAGCGAA ACCACAGCCA AGGGAACGGG CTTGGCGGAA TCAGCGGGGA AAGAAGACCC TGTTGAGCTT
3901 GACTCTAGTC TGGCACGGTG AAGAGACATG AGAGGTGTAG AATAAGTGGG AGGCCCCCGG CGCCCCCCCG GTGTCCCCGC GAGGGGCCCG GGGCGGGGTC
4001 CGCGGCCCTG CGGGCCGCCG GTGAAATACC ACTACTCTGA TCGTTTTTTC ACTGACCCGG TGAGGCGGGG GGGCGAGCCC GAGGGGCTCT CGCTTCTGGC
4101 GCCAAGCGCC CGCCCGGCCG GGCGCGACCC GCTCCGGGGA CAGTGCCAGG TGGGGAGTTT GACTGGGGCG GTACACCTGT CAAACGGTAA CGCAGGTGTC
H2 CAGG TGGGGAGTTT GACTGGGGCG GTACACCTGT CAAACGGT
A3 CAGG TGGGGAGTTT GACTGGGGCG GTACACCTGT CAAACGGT

4201 CTAAGGCGAG CTCAGGGAGG ACAGAAACCT CCCGTGGAGC AGAAGGGCAA AAGCTCGCTT GATCTTGATT TTCAGTACGA ATACAGACCG TGAAAGCGGG
4301 GCCTCACGAT CCTTCTGACC TTTTGGGTTT TAAGCAGGAG GTGTCAGAAA AGTTACCACA GGGATAACTG GCTTGTGGCG GCCAAGCGTT CATAGCGACG
4401 TCGCTTTTTG ATCCTTCGAT GTCGGCTCTT CCTATCATTG TGAAGCAGAA TTCGCCAAGC GTTGGATTGT TCACCCACTA ATAGGGAACG TGAGCTGGGT
4501 TTAGACCGTC GTGAGACAGG TTAGTTTTAC CCTACTGATG ATGTGTTGTT GCCATGGTAA TCCTGCTCAG TACGAGAGGA ACCGCAGGTT CAGACATTTG
4601 GTGTATGTGC TTGGCTGAGG AGCCAATGGG GCGAAGCTAC CATCTGTGGG ATTATGACTG AACGCCTCTA AGTCAGAATC CCGCCCAGGC GAACGATACG
4701 GCAGCGCCGC GGAGCCTCGG TTGGCCTCGG ATAGCCGGTC CCCCGCCTGT CCCCGCCGGC GGGCCGCCCC CCCCTCCACG CGCCCCGCCG CGGGAGGGCG
4801 CGTGCCCCGC CGCGCGCCGG GACCGGGGTC CGGTGCGGAG TGCCCTTCGT CCTGGGAAAC GGGGCGCGGC CGGAAAGGCG GCCGCCCCCT CGCCCGTCAC
4901 GCACCGCACG TTCGTGGGGA ACCTGGCGCT AAACCATTCG TAGACGACCT GCTTCTGGGT CGGGGTTTCG TACGTAGCAG AGCAGCTCCC TCGCTGCGAT
5001 CTATTGAAAG TCAGCCCTCG ACACAAGGGT TTGTC

Supplemental Table S3 Oligonucleotides used for Mature miRNA Quantitation

	primers<br>for linker ligation only	for linker ligation and stem-loops	for stem loops only	sequence	Stem looped primer
let-7c		hsa-let7c_15		TGAGGTAGTAGGTTG	GTCGTATCCAGTGCAGGGTCCGAGGTATTCGCACTGGATACGACAACC
let-7f	hsa-let7f_14			TGAGGTAGTAGATTG	N/A
let-7g		hsa-let7g_16		TGAGGTAGTAGTTTGT	GTCGTATCCAGTGCAGGGTCCGAGGTATTCGCACTGGATACGACAACT
miR-107		hsa-miR107_15		AGCAGCATTGTACAG	GTCGTATCCAGTGCAGGGTCCGAGGTATTCGCACTGGATACGACtgatag
miR-1246		hsa-miR1246_16		AATGGATTTTTGGAGC	GTCGTATCCAGTGCAGGGTCCGAGGTATTCGCACTGGATACGACCCTG
miR-1275	hsa-miR-1275_14			GTGGGGGAGAGGCT	N/A
miR-1275			hsa-miR-1275_15*	GTGGGGGGAGAGGCT	GTCGTATCCAGTGCAGGGTCCGAGGTATTCGCACTGGATACGACGACA
miR-148a			miR-148a_15	tcagtgcactacagaa	GTCGTATCCAGTGCAGGGTCCGAGGTATTCGCACTGGATACGACacaaa
miR-149*		hsa-miR149*_15		AGGGAGGGACGGGGG	GTCGTATCCAGTGCAGGGTCCGAGGTATTCGCACTGGATACGACCTCC
miR-16		hsa-miR16_14		TAGCAGCACGTAAA	GTCGTATCCAGTGCAGGGTCCGAGGTATTCGCACTGGATACGACcgcca[illegible]
miR-1915	hsa-miR1915_15			CCCCAGGGCGACGCG	N/A
miR-196a			miR-196a_15	taggtagtttcatgt	GTCGTATCCAGTGCAGGGTCCGAGGTATTCGCACTGGATACGACcccaa[illegible]
miR-200b			miR-200b_15	taatactgcctggta	GTCGTATCCAGTGCAGGGTCCGAGGTATTCGCACTGGATACGACtcatca
miR-200c	hsa-miR200c_14			TAATACTGCCGGGT	N/A
miR-21		hsa-mir21_15		TAGCTTATCAGACTG	GTCGTATCCAGTGCAGGGTCCGAGGTATTCGCACTGGATACGACtcaaca
miR-210			miR-210_15	ctgtgcgtgtgacag	GTCGTATCCAGTGCAGGGTCCGAGGTATTCGCACTGGATACGACtcagc[illegible]
miR-22	hsa-miR-22_14			AAGCTGCCAGTTGA	N/A
miR-23a		hsa-miR23a_14		ATCACATTGCCAGG	GTCGTATCCAGTGCAGGGTCCGAGGTATTCGCACTGGATACGACGGAA
miR-27b		hsa-miR27b_14-5		CAGTGGCTAAGTTC	N/A
miR-30c	hsa-miR30c_15			TGTAAACATCCTACA	N/A
miR-335			miR-335_15	tcaagagcaataacg	GTCGTATCCAGTGCAGGGTCCGAGGTATTCGCACTGGATACGACacattt
miR-345	hsa-miR345_15			GCTGACTCCTAGTCC	N/A
miR-373			miR-373_15	gaagtgcttcgattt	GTCGTATCCAGTGCAGGGTCCGAGGTATTCGCACTGGATACGACacacc[illegible]
miR-451		hsa-miR451_16		AAACCGTTACCATTAC	GTCGTATCCAGTGCAGGGTCCGAGGTATTCGCACTGGATACGACAACT
miR-483-5P		hsa-miR483-5p_15		AAGACGGGAGGAAAG	GTCGTATCCAGTGCAGGGTCCGAGGTATTCGCACTGGATACGACCTCC
miR-520c			miR-520c_15	aaagtgcttcctttt	GTCGTATCCAGTGCAGGGTCCGAGGTATTCGCACTGGATACGACaccct[illegible]
miR-638	hsa-miR638_15			AGGGATCGCGGGCGG	N/A
miR-720		hsa-miR720_15		TCTCGCTGGGGCCTC	GTCGTATCCAGTGCAGGGTCCGAGGTATTCGCACTGGATACGACTGGA
miR-99a		hsa-miR100_15		AACCCGTAGATCCGA	GTCGTATCCAGTGCAGGGTCCGAGGTATTCGCACTGGATACGACcacaa
MODBAN	3'modban_rcrtPCR			ATTGATGGTGCCTACAG	

Supplemental Table S4 Stem Looped Primers used for Simultaneous Analyses of Multiple Transcripts

STEM-LOOPED PRIMER AMPLIFICATION

hsa-	MIRNA SEQUENCE	PRIMER NAME	PRIMER SEQUENCE
miR-16	UAGCAGCACGUAAAUA*UUGGCG*	hsa-miR-16-14	TAGCAGCACGTAAA
		miR-16_14-loli	GTCGTATCCAGTGCAGGGTCCGAGGTATTCGCACTGGATACGAC*CGCCAA*
miR-1246	AAUGGAUUUUUGG*AGCAGG*	1246_loli	GTCGTATCCAGTGCAGGGTCCGAGGTATTCGCACTGGATACGAC*CCTGCT*
		1246_16	AATGGATTTTTGGAGC
miR-720	UCUCGCUGGGG*CCUCCA*	720_loli	GTCGTATCCAGTGCAGGGTCCGAGGTATTCGCACTGGATACGAC*TGGAGG*
		720_14	CTCGCTGGGGCCTC
miR-451	AAACCGUUACCAUUAC*UGAGUU*	451_loli	GTCGTATCCAGTGCAGGGTCCGAGGTATTCGCACTGGATACGAC*AACTCA*
		hsa-miR451_15	AAACCGTTACCATTA
let-7g	UGAGGUAGUAGUUUGU*ACAGUU*	let-7g-loli	GTCGTATCCAGTGCAGGGTCCGAGGTATTCGCACTGGATACGAC*AACTGT*
		hsa-let-7g-16	TGAGGTAGTAGTTTGT
miR-99A (miR-100)	AACCCGUAGAUCCGAU*CUUGUG*	miR-100-loli	GTCGTATCCAGTGCAGGGTCCGAGGTATTCGCACTGGATACGAC*CACAAG*
		100-15	AACCCGTAGATCCGA
miR-210	CUGUGCGUGUGACAGC*GGCUGA*	miR-210-loli	GTCGTATCCAGTGCAGGGTCCGAGGTATTCGCACTGGATACGAC*TCAGCC*
		miR-210_15	CTGTGCGTGTGACAG
internal loading control:			
INT-RNA	GGAAAGGGGAACCCCUGUGUGU	INT_RNA-loli	GTCGTATCCAGTGCAGGGTCCGAGGTATTCGCACTGGATACGAC*ACACAC*
		INT_15	GGAAAGGGGAACCCC
		INT_RNA	GGGGGGAAAAACCCCUUUUUCC
miR-1275	GUGGGGGAGAG*GCUGUC*	1275_loli	GTCGTATCCAGTGCAGGGTCCGAGGTATTCGCACTGGATACGAC*GACAGC*
		hsa-miR-1275_15*	GTGGGGGGAGAGGCT
lolirev		loli_rev	GTGCAGGGTCCGAGGT

Supplemental Table S5. Confirmed sequences of qPCR products quantified using linker ligated primers

**hsa-miR-221**	**AGCUACAUUGUCUGCTGGGUUUC**
1_B05_271599_ECRC	AGCTACATTGTCTGCTGGGTTTC
1_F03_271602_CCRC	AGCTACATTGTCTGCTGGGTTTC
1_B04_271597_EA	AGCTACATTGTCTGCTGGGTTTC
1_F02_271600_CA	AGCTACATTGTCTGCTGGGTT**A**C
1_F04_271598_EBRC	AGCTACATTGTCTGCTGGGTTTC

**hsa-miR-222**	**AGCUACAUCUGGCUACUGGGU**
1_C04_271603_EA	AGCTACATCTGGCTACTGGGT
1_G04_271604_EB	AGCTACATCTGGCTACTGGGT
1_G02_271606_CA	AGCTACATCTGGCTACTGGGT
1_C03_271607_CB	AGCTACATCTGGCTACTGGGT
1_G03_271608_CC	AGCTACATCTGGCTACTGGGT

**hsa-miR-100**	**AACCCGUAGAUCCGAACUUGUG**
**hsa-miR-99a**	**AACCCGUAGAUCCGAUCUUGUG**
1_A04_271609_EA	AACCCGTAGATCCGA**T**CTTGT
1_E03_271614_CC	AACCCGTAGATCCGA**T**CTTGTG
1_E02_271612_CA	AACCCGTAGATCCGA**T**CTTGTG
1_A03_271613_CB	AACCCGTAGATCCGA**T**CTTGT
1_A05_271611_EC	AACCCGTAGATCCGA**T**CTTGTG

**hsa-miR-720**	**UCUCGCUGGGGCCUCCA**
1_D04_271615_EA	T**A**TCGCTGGGGCCTCCA
1_D05_271617_EC	TCTCGCTGGGGCCTCCA
1_H04_271616_EB	TCTCGCTGGGGCCTCCA
1_D03_271619_CB	TCTCGCTGGGGCCTCCA
1_H02_271618_CArc	TCTCGCTGGGGCCTCCA

Confirmed sequences for looped miRs

**hsa-miR-16**	**UAGCAGCACGUAAAUAUUGGCG**
1_A09_2742	TAGCAGCACGTAAATATTGGCG
1_B09_2742	TAGCAGCACGTAAATATTGGCG
1_H11_2742	TAGCAGCACGTAAATATTGGCG

**hsa-miR-1246**	**AAUGGAUUUUUGGAGCAGG**
1_E07_2742rc	AATGGATTTTTGGAGCAGG
1_F07_2742rc	AATGGATTTTTGGAGCAGG
1_G07_2742rc	AATGGATTTTTGGAGCAGG
1_H07_2742	AATGGATTTTTGGAGCAGG

**hsa-miR-720**	**UCUCGCUGGGGCCUCCA**
1_H10_2742	TCTCGCTGGGGCCTCCA
1_B11_2742	TCTCGCTGGGGCCTCCA
1_C11_2742	TCTCGCTGGGGCCTCCA

**hsa-miR-451**	**AAACCGUUACCAUUACUGAGUU**
1_B10_2742 rc	AAACCGTTACCATTACTGAG--
1_A10_2742 rc	AAACCGTTACCATTACTGAGTT
1_C10_2742	AAACCGTTACCATTACTGAGTT

**hsa-let-7g**	**UGAGGUAGUAGUUUGUACAGUU**
1_D11_2742	TGAGGTAGTAGTTTGTACAGTT
1_F11_2742	TGAGGTAGTAGTTTGTACAGTT

**>hsa-miR-1275**	**GUGGGGGAGAGGCUGUC**
1_E08_2742 Trc	GTGGGGGAGAGGCTGTC
1_C08_2742 Lrc	GTGGGGGAGAGGCTGTC
1_H08_2742 U	GTGGGGGAGAGGCTGTC
1_A08_2742 L	GTGGGGGAGAGGCTGA-

Supplemental Table S6. Primers for Quantifying pre-miRNAs and Other Immature miRNAs

RNA		name	sequence
pre-let-7f	5'	let7Fm-1Fm	GATTGTATAGTTGTGGGGTAGTG
pre-let-7f	3'	let7Rm-1Rm	GGGAAGGCAATAGATTGTATAG
pre-let-7g	5'	let7gFm	GTAGTAGTTTGTACAGTTTGAGGGT
pre-let-7g	3'	let7gRm	GGCAGTGGCCTGTACAGT
pre-let-7c	5'	let7cFm	TTGAGGTAGTAGGTTGTATGGTT
pre-let-7c	3'	let7cRm	GGAAAGCTAGAAGGTTGTACAG
pre-let-7c	pri-S	Let-7c-US1	TGAAGCAACATTGGAAGC
pre-let-7c	pri-L	Let-7c-US2	TCTATATCCTTGCCAAGCC
pre-miR-100	5'	miR-100Fm	AACCCGTAGATCCGAACTTG
pre-miR-100	3'	miR-100Rm	TACCTATAGATACAAGCTTGTGCG
pre-miR-1246	5'	hsa-miR1246_19	TTGAATGGATTTTTGGAGC
pre-miR-1246	3'	1246_matrev_22	ATTGCTAGCCTATGGATTGATT
pre-miR-1275	5'	hsa-miR-1275_15*	GTGGGGGGAGAGGCT
pre-miR-1275	3'	1275_mat rev	TCCCTCTGCCTTGGG
pre-miR-149*	5'	149*_matfor	GCCGGCGCCCGAGCT
pre-miR-149*	3'	149*_matrev	GCCCCCGTCCCTCCC
pre-miR-155	5'	miR-155Fm	GTTAATGCTAATCGTGATAGGG
pre-miR-155	3'	miR-155Rm	GCTAATATGTAGGAGTCAGTTGGA
pre-miR-16	5'	miR-016-1Fmm	GCAGCACGTAAATATTGGCGT
pre-miR-16	3'	miR-016-1Rm	CAGCAGCACAGTTAATACTGGAGA
pre-miR-1915	5'	1915_matfor	TGAGAGGCCGCACCT
pre-miR-1915	3'	1915_matrev_13	CGCGTCGCCCTGG
pre-miR-200c	5'	miR-200cFm	CTCGTCTTACCCAGCAGTGT
pre-miR-200c	3'	miR-200cRm2	CATCATTACCCGGCAGTATTAG
pre-miR-200c	pri-S	miR-200c US1	GAAGCTGCCTGACCCAAG
pre-miR-200c	pri-L	miR-200c US2	CAGGGATCTGCAGCTTTTC
pre-miR-221	5'	miR-221Fm	CCTGGCATACAATGTAGATTTCTG
pre-miR-221	3'	miR-221Rm	AAACCCAGCAGACAATGTAGCT
pre-miR-221	pri-S	miR-221 US1	AAATAGTATGTGAGAATTACTTGCAAGC
pre-miR-221	pri-L	miR-221 US2	GAAATTTTGTTGGTAGTAGGTAAGTCC
pre-miR-222	5'	miR-222(p)Fm	CCCCAGAAGGCAAAGGAT
pre-miR-222	3'	miR-222(p)Rm	CTCTCTCAGGACACTGAAGCAG
pre-miR-23a	5'	miR-023aFm	CTGGGGTTCCTGGGGAT
pre-miR-23a	3'	miR-023aRm	TGGTAATCCCTGGCAATGTG
pre-miR-27	5'	miR-027a,bFm	GCAGGGCTTAGCTGCTTG
pre-miR-27	3'	miR-027a,bRm	GGCGGAACTTAGCCACTGT
pre-miR-30c	5'	miR-030c-1,2Fm	TGTGTAAACATCCTACACTCTCAG
pre-miR-30c	3'	miR-030c-1,2Rm	GAGTAAACAACCCTCTCCCA
pre-miR-451	5'	hsa-miR-451_21	CAAGGAAACCGTTACCATTAC
pre-miR-451	3'	451_mat rev	TCTGGGTATAGCAAGAGAACC
pre-miR-638	5'	638_13	AGGGATCGCGGGC
pre-miR-638	3'	638_matrev	AGCGCCGCAGTTACG
pre-pre-miR-21	5'	miR-021Fm	GCTTATCAGACTGATGTTGACTG
pre-pre-miR-21	3'	miR-021Rm	CAGCCCATCGACTGGTG
pre-pre-miR-21	pri-S	miR-021 US1	ATCTCCATGGCTGTACCACC
pre-pre-miR-21	pri-L	miR-021 US2	TCAAATCCTGCCTGACTGTC

Supplemental Table S7 Sequences of Immature miRNAs Detected

```
           1                                                                                                                    110
hsa-mir-221UGAACAUCCA GGUCUGGGGC AUGAACCUGG CAUACAAUGU AGAUUUCUGU GUUCGUUAGG CAACAGCUAC AUUGUCUGCU GGGUUUCAGG CUACCUGGAA ACAUGUUCUC
Cmim221C_1                            CCTGG CATACAATGT AGATTTCTGT GTTCGTTAGG CAACAGCTAC ATTGTCTGCT GGGTTT
Cmim221B_1                            CCTGG CATACAATGT AGATTTCTGT GTTCGTTAGG CAACAGCTAC ATTGTCTGCT GGGTTT
Emrcm221C_                            CCTGG CATACAATGT AGATTTCTGT GTTCGTTAGG CAACAGCTAC ATTGTCTGCT GGGTTT
Crcim221A_                            CCTGG CATACAATGT AGATTTCTGT GTTCGTTAGG CAACAGCTAC ATTGTCTGCT GGGTTT
Emim221A_1                            CCTGG CATACAATGT AGATTTCTGT GTTCGTTAGG CAACAGCTAC ATTGTCTGCT GGGTTT
Emim221B_1                                                  CGCGT CGCCCTGGGC AGAAATCTAC ATTGTATGCC AGG
```

```
               1                                                                            72
hsa-mir-21     UGUCGGGUAG CUUAUCAGAC UGAUGUUGAC UGUUGAAUCU CAUGGCAACA CCAGUCGAUG GGCUGUCUGA CA
Cmim21B_16              G CTTATCAGAC TGATGTTGAC TGTTGAATCT CATGGCAACA CCAGTCGATG GGCTG
Crcim21C_1              G CTTATCAGAC TGATGTTGAC TGTTGAATCT CATGGCAACA CCAGTCGATG GGCTG
Ercim21B_1              G CTTATCAGAC TGATGTTGAC TGTTGAATCT CATGGCAACA CCAGTCGATG GGCTG
Crcim21A_1              G CTTATCAGAC TGATGTTGAC TGTTGAATCT CATGGCAACA CCAGTCGATG GGCTG
Ercim21C_1              G CTTATCAGAC TGATGTTGAC TGTTGAATCT CATGGCAACA CCAGTCGATG GGCTG
Ercim21A_1              G CTTATCAGAC TGATGTTGAC TGTTGAATCT CATGGCAACA CCAGTCGATG GGCTG
```

```
                1                                                           73
hsa-miR-23a     GGCCGGCUG GGGUUCCUGG GGAUGGGAUU UGCUUCCUGU CACAAAUCAC AUUGCCAGGG AUUUCCAACC GACC
CC23a_186             CTG GGGTTCCTGG GGATGGGATT TGCTTCCTGT CACAAATCAC ATTGCCAGGG ATTACCA
EC23a_183             CTG GGGTTCCTGG GGATGGGATT TGCTTCCTGT CACAAATCAC ATTGCCAGGG ATTACCA
EB23a_182             CTG GGGTTCCTGG GGATGGGATT TGCTTCCTGT CACAAATCAC ATTGCCAGGG ATTACCA
EA23a_181             CTG GGGTTCCTGG GGATGGGATT TGCTTCCTGT CACAAATCAC ATTGCCAGGG ATTACCA
CB23a_185             CTG GGGTTCCTGG GGATGGGATT TGCTTCCTGT CACAAATCAC ATTGCCAGGG ATTACCA
CA23a_184             CTG GGGTTCCTGG GGATGGGATT TGCTTCCTGT CACAAATCAC ATTGCCAGGG ATTACCA
```

```
               1                                                                                              90
hsa-mir-161    GUCAGCAGUG CCUUAGCAGC ACGUAAAUAU UGGCGUUAAG AUUCUAAAAU UAUCUCCAGU AUUAACUGUG CUGCUGAAGU AAGGUUGAC
Crcim16C_1                     GCAGC ACGTAAATAT TGGCGTTAAG ATTCTAAAAT TATCTCCAGT ATTAACTGTG CTGCTG
Cfcim16B_1*                    GCAGC ACGTAAATAT TGGCGTTAAG ATTCTAAAAT TATCTCCAGT ATTAACTGTG CTGCTG
Efcim16A_1*                    GCAGC ACGTAAATAT TGGCGTTAAG ATTCTAAAAT TATCTCCAGT ATTAACTGTG CTGCTG
Efcim16B_1*                    GCAGC ACGTAAATAT TGGCGTTAAG ATTCTAAAAT TATCTCCAGT ATTAACTGTG CTGCTG
Cfcim16A_1*                    GCAGC ACGTAAATAT TGGCGTTAAG ATTCTAAAAT TATCTCCAGT ATTAACTGTG CTGCTG
```

miR-200c

```
immature      CAGGGAUCU GCAGCUUUUC CGCAGGGAUC CUGGGCCUGA AGCUGCCUGA CCCAAGGUGG GCGGGCUGGG CGGGGGCCCU CGUCUUACCC AGCAGUGUUU GGGUGCGGUU GGGAGUCUCU AAUACUGCCG
                    GGUAAUGAUG GAGG
EfiUS2C       CAGGGATCT GCAGCTTTTC CGCAGGGATC CTGGGCCTGA AGCTGCCTGA CCCAAGGTGG GCGGGCTGGG CGGGGGCCCT CGTCTTACCC AGCAGTGTTT GGGTGCGGTT GGGAGTCTCT AATACTGCCT
                    GGTAATGATG AC
EriUS2B       CAGGGATCT GCAGCTTTTC CGCAGGGATC CTGGGCCTGA AGCTGCCTGA CCCAAGGTGG GCGGGCTGGG CGGGGGCCCT CGTCTTACCC AGCAGTGTTT GGGTGCGGTT GGGAGTCTCT AATACTGCCT
                    GGTAATGATG AC
CriUS1B                                              TGA AGCTGCCTGA CCCAAGGTGG GCGGGCTGGG CGGGGGCCCT CGTCTTACCC AGCAGTGTTT GGGTGCGGTT GGGAGTCTCT AATACTGCCT
                    GGTAATGATG AC
CfiUS1C                                               GA AGCTGCCTGA CCCAAGGTGG GCGGGCTGGG CGGGGGCCCT CGTCTTACCC AGCAGTGTTT GGGTGCGGTT GGGAGTCTCT AATACTGCCT
                    GGTAATGATG AC
CfiUS1A                                               GA AGCTGCCTGA CCCAAGGTGG GCGGGCTGGG CGGGGGCCCT CGTCTTACCC AGCAGTGTTT GGGTGCGGTT GGGAGTCTCT AATACTGCCT
                    GGTAATGATG AC
Cri200cC                                                                                        CT CGTCTTACCC AGCAGTGTTT GGGTGCGGTT GGGAGTCTCT AATACTGCCT
                    GGTAATGATG AC
Cri200cA                                                                                        CT CGTCTTACCC AGCAGTGTTT GGGTGCGGTT GGGAGTCTCT AATACTGCCT
                    GGTAATGATG AC
Efi200cA_                                                                                       CT CGTCTTACCC AGCAGTGTTT GGGTGCGGTT GGGAGTCTCT AATACTGCCT
                    GGTAATGATG AC   Efi200cB_                                                                                                 CT CGACTTACCC
                    AGCAGTGTTT GGGTGCGGTT GGGAGTCTCT AATACTGCCT GGTAATGATG AC
```

```
            1                                                                                        84
hsa-let-7g  AGGCUGAGGU AGUAGUUUGU ACAGUUUGAG GGUCUAUGAU ACCACCCGGU ACAGGAGAUA ACUGUACAGG CCACUGCCUU GCCA
Crcim7gC_1          GT AGTAGTTTGT ACAGTTTGAG GGTCTATGAT ACCACCCGGT ACAGGAGATA ACTGTACAGG CCACTGCC
Crcim7gB_1          GT AGTAGTTTGT ACAGTTTGAG GGTCTATGAT ACCACCCGGT ACAGGAGATA ACTGTACAGG CCACTGCC
Emim7gA_15          GT AGTAGTTTGT ACAGTTTGAG GGTCTATGAT ACCACCCGGT ACAGGAGATA ACTGTACAGG CCACTGCC
Cmim7gA_16          GT AGTAGTTTGT ACAGTTTGAG GGTCTATGAT ACCACCCGGT ACAGGAGATA ACTGTACAGG CCACTGCC
```

Supplementary Figures

Supplemental Figure S1. Some Diagnostic miRNAs are Mostly Retained.

Indicated miRNAs were amplified from RNA collected from cells and the conditioned media of MCF7 cells using looped primers, and separated by native PAGE. **A** miRNAs in which the cellular (c), but not the extracellular (x) RNA population contained the indicated miRNAs. In each case of the cellular sample, the main band (arrow) was excised, cloned and sequenced and found to be the expected amplified product. In the case of extracellular samples the main bands, all of which migrated differently (star), had no resemblance to the miRNA to be amplified as determined by sequencing. **B** miRNAs in which the released miRNA population contained a band of much lower abundance than the retained population.

Supplemental Figure S2. Lack of Correlation between Primary Transcript and Release Rate of MiRNAs. Released/retained miRNAs (x/c) as evaluated in Figure 1 are plotted according to chromosomal location (chromosomes 1-X) from top to bottom. Micro-RNA clusters are indicated in color. Note that for cluster miR-200c-141, which is located in a single intron (1), 30 times more of its encoded miR-200c molecules than miR-141 molecules were released from cells (**Figure 2A,** and data not shown) than retained, indicating that the extent of miRNA release is not determined at the primary-miRNA level. In support of this idea, within the other miR-200 cluster, which encodes miR-200a, miR-200b and miR-429 (1), more than half of miR-429 and miR-200b molecules are released, whereas most of miR-200a is retained. Therefore, the extracellular accumulation of mature miRNAs is regulated at levels other than the primary transcript abundance. MicroRNAs contained in particular clusters are indicated by red or green colored bars.

Supplemental Figure S3. MiR-1246 is a Reliable Indicator of Body Fluids Conditioned by Mammary Epithelia. A tumor growth of xenografted cells of mice used for assessment of miRNA abundance in blood plasma as indicated in Figure 4A. The tumor size is presented as the product of the

3 diameters. Data presented in Figure 6 are from bleeds at day 30 (arrow). **B** PAGE of end-point PCR of miR-1246 of bleeds of MCF7-1 and MDA-MB-231.

Supplemental Figure S4. Fetal Bovine Serum Does Not Interfere With Extracellular MiRNA Assessment. A Relative abundance of indicated miRNAs in calf serum (CS) and fetal bovine serum (FBS), normalized to INT-RNA**.** Note the absence of miR-451 from FBS, but that CS contains measurable levels of *Bos taurus* miR-451, which differs from hsa-miR-451 by a single terminal nucleotide (2). **B** End-point PCR using stem-loop primers on extracellular (x) and cellular (c) miR-451 of indicated breast cancer cell lines grown in complete FBS, or in FBS depleted of microvesicles (FBS S100). **C** Ratio of miRNAs in c and x of cells grown in complete FBS and FBS depleted of microvesicles (FBS S100). **D.** MiRNAs measured as in Supplemental Figure S4C, by qRT-PCR using linker-ligation. Error bars indicate standard deviation. A is an average of 2 experiments, C and E are averages of 3 independent experiments.

Supplemental Figure S5. Template-independent amplification products at low template concentrations. Mature miR-16 was assessed by the stem-loop-primer protocol on 5-fold serial dilutions of a synthetic DNA construct reflecting the expected product of miR-16.

References of Supplementary Material

1. Bracken CP, Gregory PA, Kolesnikoff N, Bert AG, Wang J, Shannon MF, Goodall GJ. A double-negative feedback loop between ZEB1-SIP1 and the microRNA-200 family regulates epithelial-mesenchymal transition. Cancer Res. 2008 Oct 1;68(19):7846-54.
2. Long JE, Chen HX. Identification and characteristics of cattle microRNAs by homology searching and small RNA cloning. Biochem Genet. 2009 Jun;47(5-6):329-43.

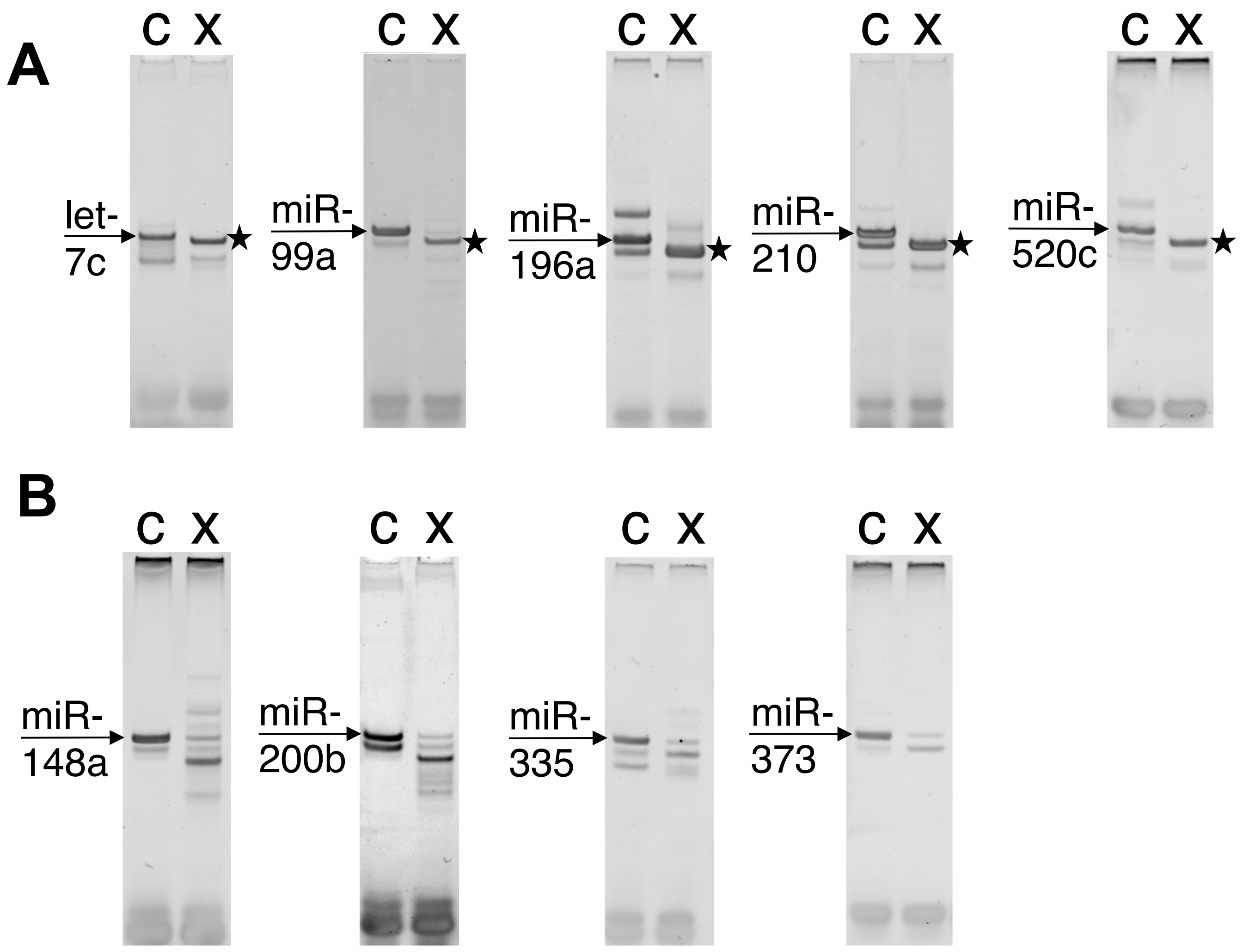

Pigati et al. Supplemental Figure S1

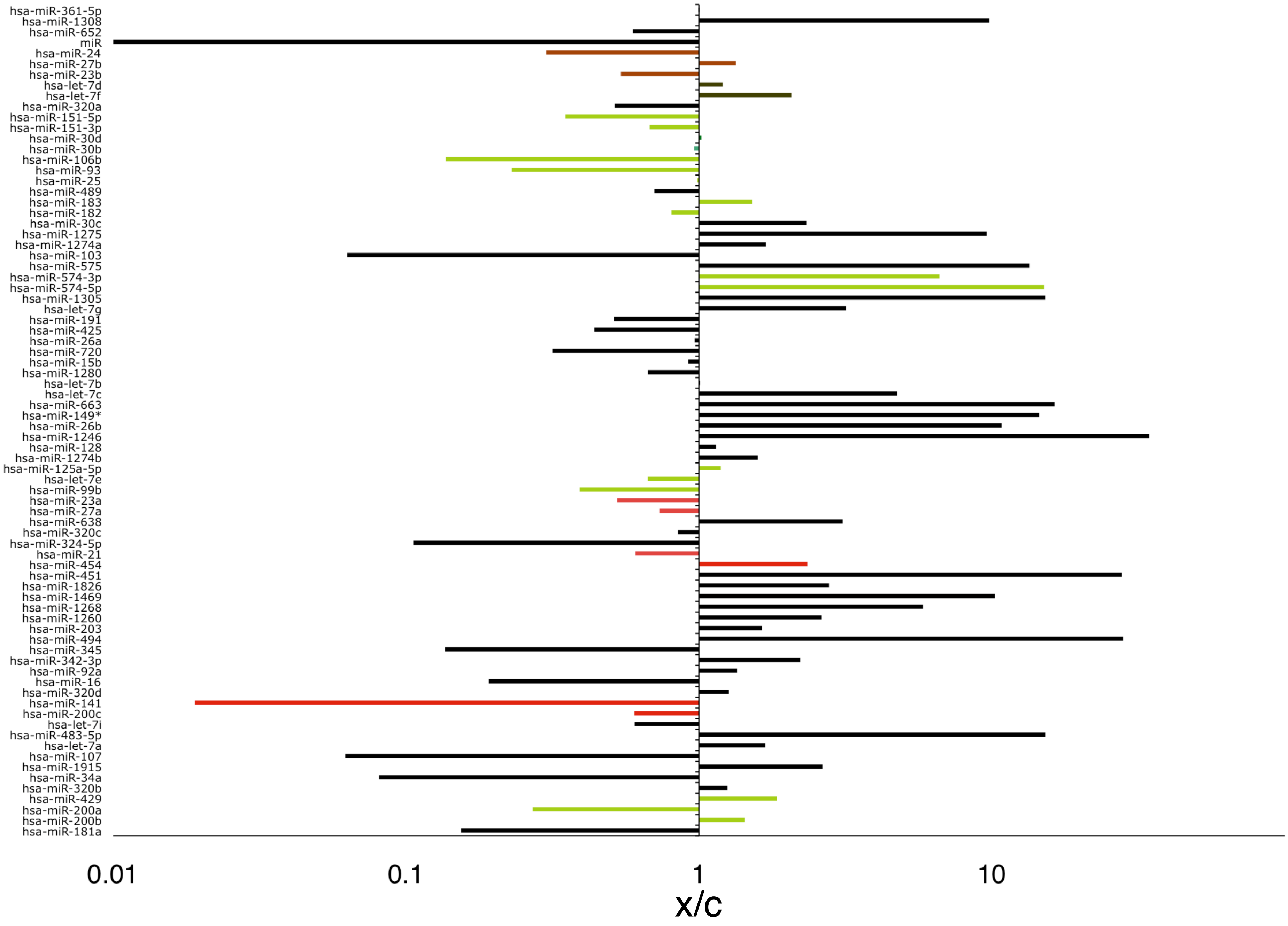

Pigati et al. Supplemental Figure S2

A

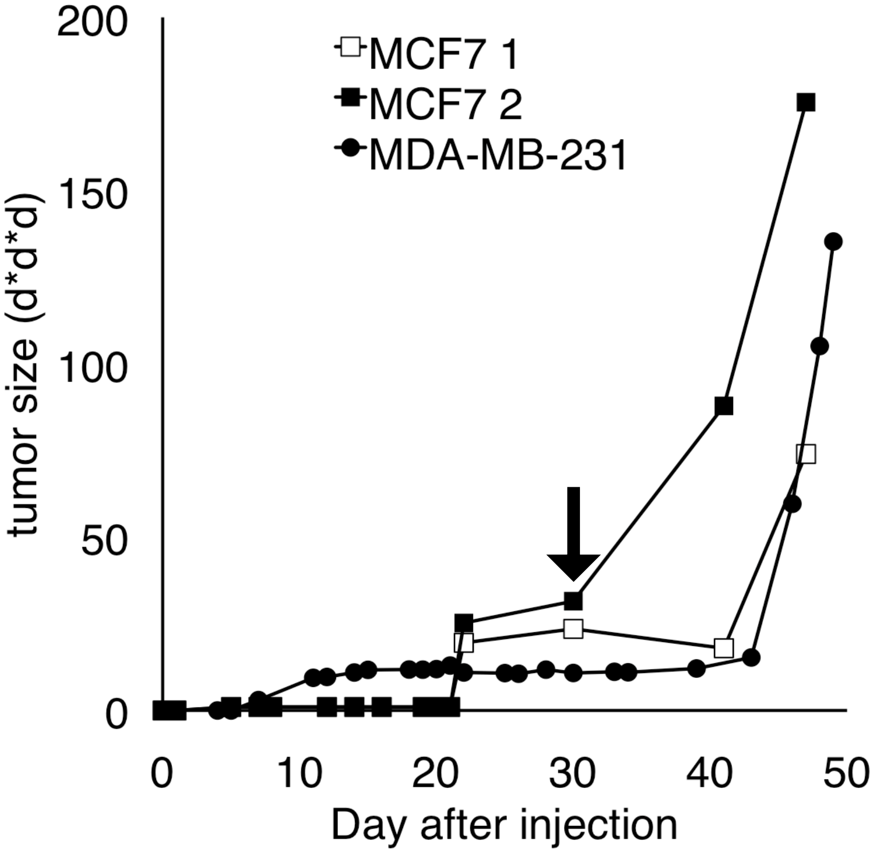

B

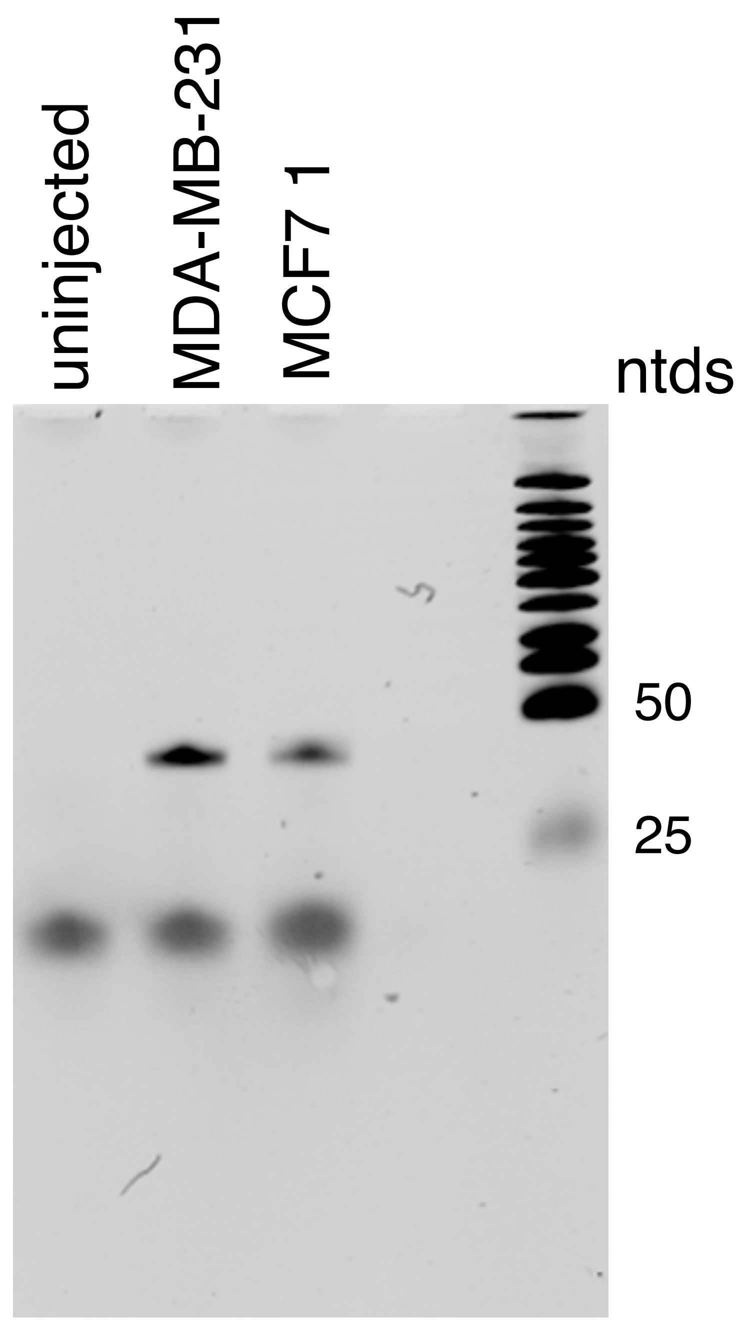

Pigati et al. Supplemental Figure S3

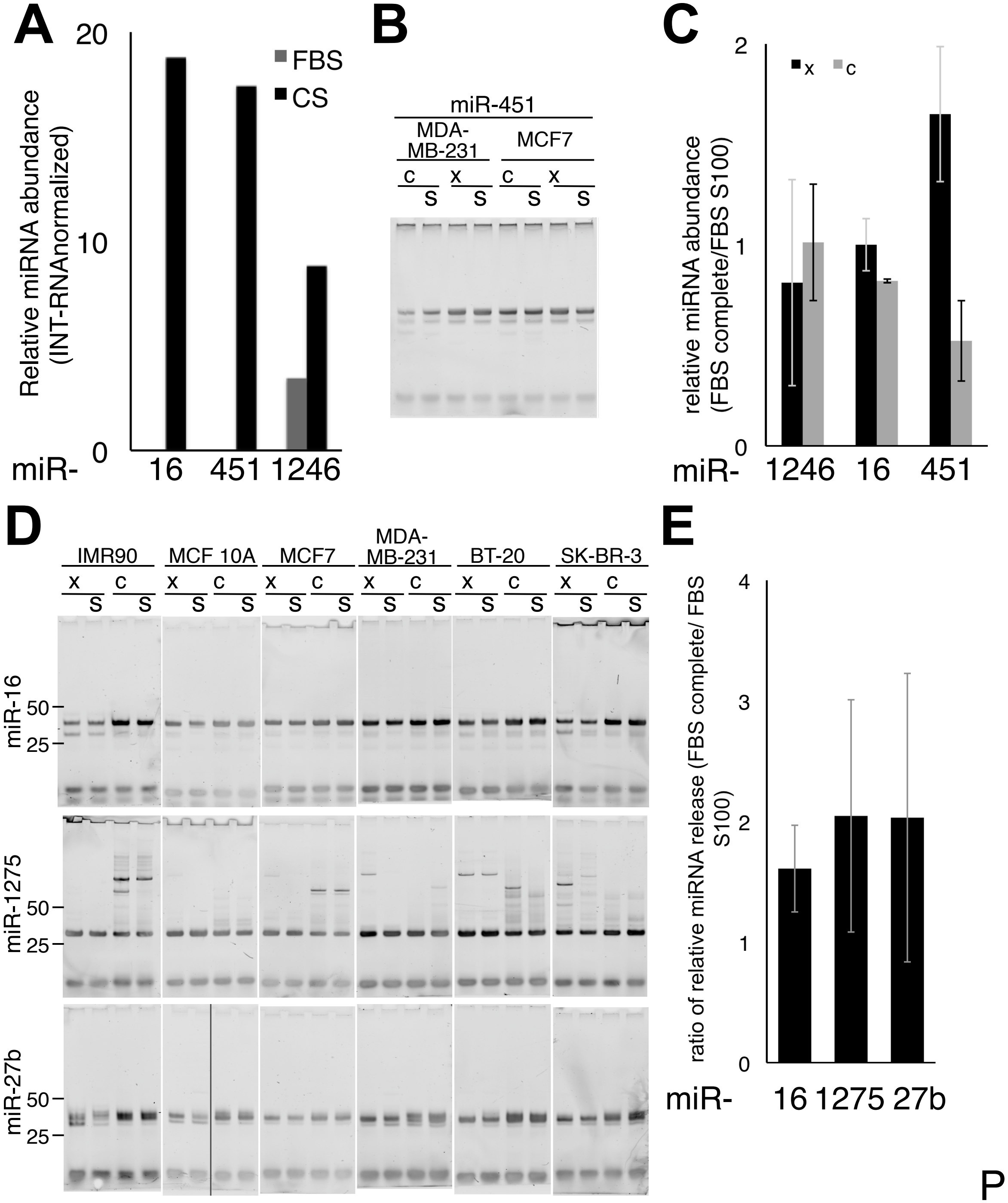

Pigati et al. Supplemental Figure S4

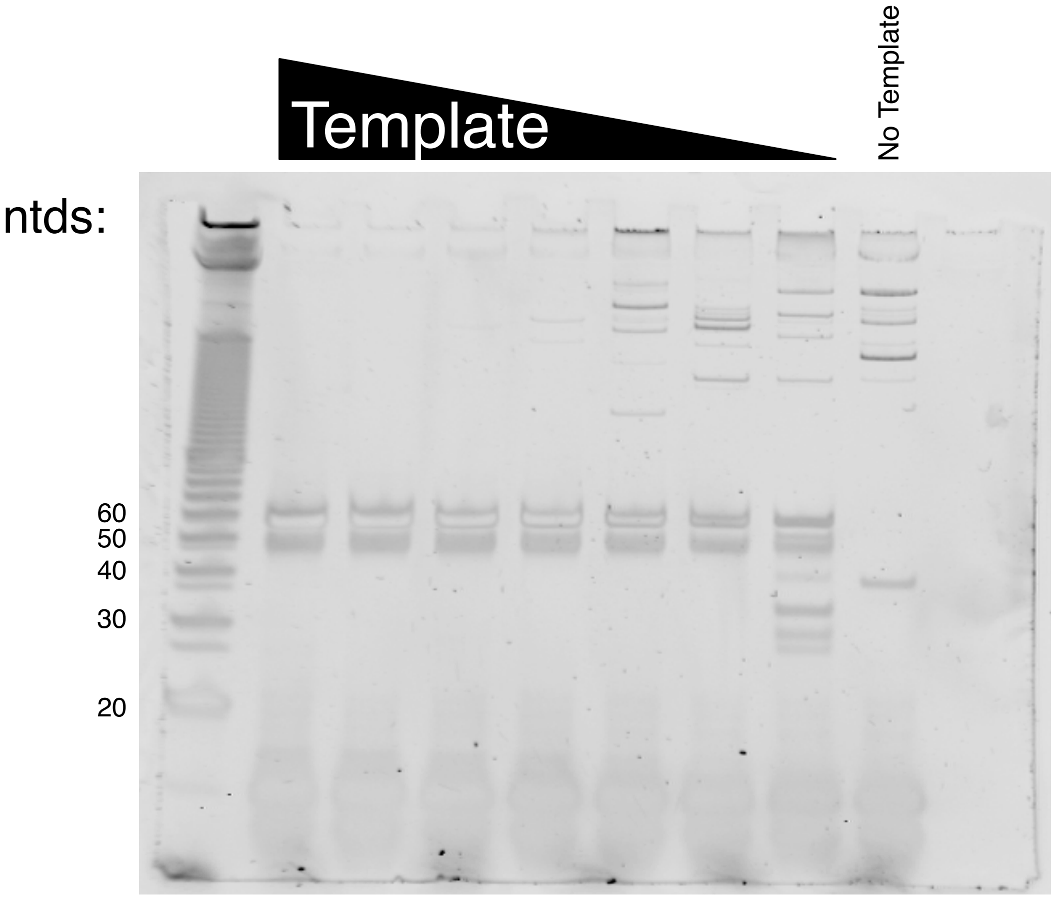

Pigati et al. Supplemental Figure S5
