# Supplementary material for: Selective Release of MicroRNA Species from Normal and Malignant Mammary Epithelial Cells
Source: PLoS One. 2010 Oct 20;5(10):e13515. doi: 10.1371/journal.pone.0013515 (PMC2958125; doi:10.1371/journal.pone.0013515)
Supplement: Table S1 — RRNA and snRNA fragments detected in unique RNA band of extracellular miRNAs. RNA sequences cloned and sequenced from bands in Figure 1, D and E, marked with a star. (0.03 MB DOC) [file pone.0013515.s006.doc]

**Table S1A**

**Hs 5.8S ribosomal RNA**

1 GACTCTTAGC GGTGGATCAC TCGGCTCGTG CGTCGATGAAG AACGCAGCTAG

51 CTGCGAGAAT TAATGTGAAT TGCAGGACAC ATTGATCATCG ACACTTCGAAC

101 GCACTTGCGG CCCCGGGTTC CTCCCGGGGC TACGCCTGTCT GAGCGTCGCTT

**2_B1 TTTTTTTTTT TTTTTTTTTT CTCCCGGGGC TACGCCTGTCT GAGCGTCGC**

**1_B2 TTTTTTTTTT TTTTTTTTTT CTCCCGGGGC TACGCCTGTCT GAGCGTCGC**

**1_D2 TTTTTTTTTT TTTTTTTTTT CTCCCGGGGC TACGCCTGTCT GAGCGTCGC**

**2_E2 C CTCCCGGGGC TACGCCTGTCT GAGCGTCGCT**

**Table S1B**

**Hs U1A small nuclear RNA (RNU1A)**

1 ATACTTACCT GGCAGGGGAG ATACCATGAT CACGAAGGTG GTTTTCCCAG

**2_B3 TTTTTTTTTT TTTTTTTGAG ATACCATGAT CACGAAGGTG GTTTTCCCAG**

**1_E3 TTTTTTTTTT TTTTTTTGAG ATACCATGAT CACGAAGGTG GTTTTCCCAG**

51 GGCGAGGCTT ATCCATTGCA CTCCGGATGT GCTGACCCCT GCGATTTCCC

**2_B3 GGCGAGGC**

**1_E3 GGCGAGGC**

101 CAAATGTGGG AAACTCGACT GCATAATTTG TGGTAGTGGG GGACTGCGTT

151 CGCGCTTTCC CCTG
