# Supplementary material for: Selective Release of MicroRNA Species from Normal and Malignant Mammary Epithelial Cells
Source: PLoS One. 2010 Oct 20;5(10):e13515. doi: 10.1371/journal.pone.0013515 (PMC2958125; doi:10.1371/journal.pone.0013515)
Supplement: Table S2 — RNA Subpopulation Enriched in the Extracellular Space. Sequences identified with D1, D2, D3 represent sequence data of miRNAs extracted from cells, all others are miRNA sequences retrieved from the extracellular space. (0.05 MB DOC) [file pone.0013515.s007.doc]

1-5035 Homo sapiens 28S ribosomal RNA (LOC100008589), NR_003287

1 CGCGACCTCA GATCAGACGT GGCGACCCGC TGAATTTAAG CATATTAGTC AGCGGAGGAA AAGAAACTAA CCAGGATTCC CTCAGTAACG GCGAGTGAAC

**mir-923TTTTTTTTTTTTTTTTTTTTTTTTTTTTTTTTTTTTTTTTTTTTTTATTTGTC AGCGGAGGAA AAGAAACTAA CCAGGATTCC CTCAGTAATG GCGAGTG**

**G2 TTTTTTTTTTTTTTTTTTTTTTTTTTTTTTTTTTTTTTTTTTTTTTTTTTTTTTTTTTTTTTTTTTTTTTTTTTTTTTTTTTTTTTTTTTTTTTAACG GCGAGTGAAC**

**D3 TTTTTTTTTTTTTTTTTTTTTTTTTTTTTTTTTTTTTTTTTTTTTTTTTTTTTTTTTTTTTTTTTTTTTTTTTTTPTTTTTTTTTTTTTTTTTTAACG GCGAGTGAAC**

**C3 TTTTTTTTTTTTTTTTTTTTTTTTTTTTTTTTTTTTTTTTTTTTTTTTTTTTTTTTTTTTTTTTTTTTTTTTTTTPTTTTTTTTTTT AGGTAACG GCGAGTGAAC**

**D1 TTTTTTTTTTTTTTTTTTTTTTTTTTTTTTTTTTTTTTTTTTTTTTTTTTTTTTTTTTTTTTTTTTTTTTTTTTTPTTTTTTTTTTTTTTTTTTTTTTTTTTTTTGAAC**

**G1 TTTTTTTTTTTTTTTTTTTTTTTTTTTTTTTTTTTTTTTTTTTTTTTTTTTTTTTTTTTTTTTTTTTTTTTTTTTPTTTTTTTTTTTTTTTTTTTTTTTTTTTTTGAAC**

**E1 TTTTTTTTTTTTTTTTTTTTTTTTTTTTTTTTTTTTTTTTTTTTTTTTTTTTTTTTTTTTTTTTTTTTTTTTTTTPTTTTTTTTTTTTTTTTTTTTTTTTTTTTTAGGT**

101 AGGGAAGAGC CCAGCGCCGA ATCCCCGCCC CGCGGGGCGC GGGACATGTG GCGTACGGAA GACCCGCTCC CCGGCGCCGC TCGTGGGGGG CCCAAGTCCT

**G2 AGGGAAGAGC CCAGCGCCGA ATCCCCGCCC CGC**

**D3 AGGGAAGAGC CCAGCGCCGA ATCCCCGCCC CGC**

**C3 AGGGAAGAGC CCAGCGCCGA ATCCCCGCCC CGC**

**D1 AGGGAAGAGC CCAGCGCCGA ATCCCCGCCC CGC**

**G1 AGGGAAGAGC CCAGCGCCGA ATCCCCGCCC CGC**

**C2 AGGGAAGAGC CCAGCGCCGA ATCCCCGCCC CGC**

**E1 AGGGAAGAGC CCAGCGCCGA ATCCCCGCCC CGC**

**B3 TTTTTTTTTTTTTTTTTTTTTTTTTTTTTTTTTGGCGGGGCGC GGGACATGTG GCGTACGGAA GACCCGC**

**D2 TTTTTTTTTTTTTTTTTTTTTTTTTTTTTTTTTAGGGGGGCGC GGGACATGTG GCGTACGGAA GACCCGC**

201 TCTGATCGAG GCCCAGCCCG TGGACGGTGT GAGGCCGGTA GCGGCCGGCG CGCGCCCGGG TCTTCCCGGA GTCGGGTTGC TTGGGAATGC AGCCCAAAGC

301 GGGTGGTAAA CTCCATCTAA GGCTAAATAC CGGCACGAGA CCGATAGTCA ACAAGTACCG TAAGGGAAAG TTGAAAAGAA CTTTGAAGAG AGAGTTCAAG

401 AGGGCGTGAA ACCGTTAAGA GGTAAACGGG TGGGGTCCGC GCAGTCCGCC CGGAGGATTC AACCCGGCGG CGGGTCCGGC CGTGTCGGCG GCCCGGCGGA

501 TCTTTCCCGC CCCCCGTTCC TCCCGACCCC TCCACCCGCC CTCCCTTCCC CCGCCGCCCC TCCTCCTCCT CCCCGGAGGG GGCGGGCTCC GGCGGGTGCG

601 GGGGTGGGCG GGCGGGGCCG GGGGTGGGGT CGGCGGGGGA CCGTCCCCCG ACCGGCGACC GGCCGCCGCC GGGCGCATTT CCACCGCGGC GGTGCGCCGC

701 GACCGGCTCC GGGACGGCTG GGAAGGCCCG GCGGGGAAGG TGGCTCGGGG GGCCCCGTCC GTCCGTCCGT CCTCCTCCTC CCCCGTCTCC GCCCCCCGGC

**A2 GACCGGCTCC GGGACGGCTG GGAAGGCCCG GCGGGGAAGG T**

**C2 GACCGGCTCC GGGACGGCTG GGAAGGCCCG GCGGGGAAGG T**

**E2 TTTTTTTTTTTTTTTTTTTTTTTTTTTTTTTTTTTTTTTTTTTTTTTTTTTTTTTTTTTTTTTTTTTTTTTTTTTTTTTTCCTCCTC CCCCGTCTCC GCCCCCCGGC**

801 CCCGCGTCCT CCCTCGGGAG GGCGCGCGGG TCGGGGCGGC GGCGGCGGCG GCGGTGGCGG CGGCGGCGGG GGCGGCGGGA CCGAAACCCC CCCCGAGTGT

**E2 CCCGCGTCCT CCCT**

901 TACAGCCCCC CCGGCAGCAG CACTCGCCGA ATCCCGGGGC CGAGGGAGCG AGACCCGTCG CCGCGCTCTC CCCCCTCCCG GCGCCCACCC CCGCGGGGAA

**G2 CGAGGGAGCG AGACCCGTCG CCGCGCT**

1001 TCCCCCGCGA GGGGGGTCTC CCCCGCGGGG GCGCGCCGGC GTCTCCTCGT GGGGGGGCCG GGCCACCCCT CCCACGGCGC GACCGCTCTC CCACCCCTCC

1101 TCCCCGCGCC CCCGCCCCGG CGACGGGGGG GGTGCCGCGC GCGGGTCGGG GGGCGGGGCG GACTGTCCCC AGTGCGCCCC GGGCGGGTCG CGCCGTCGGG

1201 CCCGGGGGAG GTTCTCTCGG GGCCACGCGC GCGTCCCCCG AAGAGGGGGA CGGCGGAGCG AGCGCACGGG GTCGGCGGCG ACGTCGGCTA CCCACCCGAC

1301 CCGTCTTGAA ACACGGACCA AGGAGTCTAA CACGTGCGCG AGTCGGGGGC TCGCACGAAA GCCGCCGTGG CGCAATGAAG GTGAAGGCCG GCGCGCTCGC

1401 CGGCCGAGGT GGGATCCCGA GGCCTCTCCA GTCCGCCGAG GGCGCACCAC CGGCCCGTCT CGCCCGCCGC GCCGGGGAGG TGGAGCACGA GCGCACGTGT

1501 TAGGACCCGA AAGATGGTGA ACTATGCCTG GGCAGGGCGA AGCCAGAGGA AACTCTGGTG GAGGTCCGTA GCGGTCCTGA CGTGCAAATC GGTCGTCCGA

1601 CCTGGGTATA GGGGCGAAAG ACTAATCGAA CCATCTAGTA GCTGGTTCCC TCCGAAGTTT CCCTCAGGAT AGCTGGCGCT CTCGCAGACC CGACGCACCC

1701 CCGCCACGCA GTTTTATCCG GTAAAGCGAA TGATTAGAGG TCTTGGGGCC GAAACGATCT CAACCTATTC TCAAACTTTA AATGGGTAAG AAGCCCGGCT

1801 CGCTGGCGTG GAGCCGGGCG TGGAATGCGA GTGCCTAGTG GGCCACTTTT GGTAAGCAGA ACTGGCGCTG CGGGATGAAC CGAACGCCGG GTTAAGGCGC

1901 CCGATGCCGA CGCTCATCAG ACCCCAGAAA AGGTGTTGGT TGATATAGAC AGCAGGACGG TGGCCATGGA AGTCGGAATC CGCTAAGGAG TGTGTAACAA

2001 CTCACCTGCC GAATCAACTA GCCCTGAAAA TGGATGGCGC TGGAGCGTCG GGCCCATACC CGGCCGTCGC CGGCAGTCGA GAGTGGACGG GAGCGGCGGG

2101 GGCGGCGCGC GCGCGCGCGC GTGTGGTGTG CGTCGGAGGG CGGCGGCGGC GGCGGCGGCG GGGGTGTGGG GTCCTTCCCC CGCCCCCCCC CCCACGCCTC

**C3 TTTTTTTTTTTTTTTTTTTTTTTTTTTTTTTG CGTCGGAGGG CGGCGGCGGC GGCGGCGGCG GGGGT**

**2A TTTTTTTTTTTTTTTTTTTTTTTTTTTTTTTG CGTCGGAGGG CGGCGGCGGC GGCGGCGGCG GGGGT**

2201 CTCCCCTCCT CCCGCCCACG CCCCGCTCCC CGCCCCCGGA GCCCCGCGGA CGCTACGCCG CGACGAGTAG GAGGGCCGCT GCGGTGAGCC TTGAAGCCTA

2301 GGGCGCGGGC CCGGGTGGAG CCGCCGCAGG TGCAGATCTT GGTGGTAGTA GCAAATATTC AAACGAGAAC TTTGAAGGCC GAAGTGGAGA AGGGTTCCAT

2401 GTGAACAGCA GTTGAACATG GGTCAGTCGG TCCTGAGAGA TGGGCGAGCG CCGTTCCGAA GGGACGGGCG ATGGCCTCCG TTGCCCTCGG CCGATCGAAA

2501 GGGAGTCGGG TTCAGATCCC CGAATCCGGA GTGGCGGAGA TGGGCGCCGC GAGGCGTCCA GTGCGGTAAC GCGACCGATC CCGGAGAAGC CGGCGGGAGC

2601 CCCGGGGAGA GTTCTCTTTT CTTTGTGAAG GGCAGGGCGC CCTGGAATGG GTTCGCCCCG AGAGAGGGGC CCGTGCCTTG GAAAGCGTCG CGGTTCCGGC

**G3 TTTTTTTTTTTTTTTTTTTTTTTTTTTTTTTTTTTTTTTTTGT CCTGGAATGG GTTC**

2701 GGCGTCCGGT GAGCTCTCGC TGGCCCTTGA AAATCCGGGG GAGAGGGTGT AAATCTCGCG CCGGGCCGTA CCCATATCCG CAGCAGGTCT CCAAGGTGAA

2801 CAGCCTCTGG CATGTTGGAA CAATGTAGGT AAGGGAAGTC GGCAAGCCGG ATCCGTAACT TCGGGATAAG GATTGGCTCT AAGGGCTGGG TCGGTCGGGC

2901 TGGGGCGCGA AGCGGGGCTG GGCGCGCGCC GCGGCTGGAC GAGGCGCGCG CCCCCCCCAC GCCCGGGGCA CCCCCCTCGC GGCCCTCCCC CGCCCCACCC

3001 GCGCGCGCCG CTCGCTCCCT CCCCACCCCG CGCCCTCTCT CTCTCTCTCT CCCCCGCTCC CCGTCCTCCC CCCTCCCCGG GGGAGCGCCG CGTGGGGGCG

3101 CGGCGGGGGG AGAAGGGTCG GGGCGGCAGG GGCCGCGCGG CGGCCGCCGG GGCGGCCGGC GGGGGCAGGT CCCCGCGAGG GGGGCCCCGG GGACCCGGGG

3201 GGCCGGCGGC GGCGCGGACT CTGGACGCGA GCCGGGCCCT TCCCGTGGAT CGCCCCAGCT GCGGCGGGCG TCGCGGCCGC CCCCGGGGAG CCCGGCGGCG

3301 GCGCGGCGCG CCCCCCACCC CCACCCCACG TCTCGGTCGC GCGCGCGTCC GCTGGGGGCG GGAGCGGTCG GGCGGCGGCG GTCGGCGGGC GGCGGGGCGG

3401 GGCGGTTCGT CCCCCCGCCC TACCCCCCCG GCCCCGTCCG CCCCCCGTTC CCCCCTCCTC CTCGGCGCGC GGCGGCGGCG GCGGCAGGCG GCGGAGGGGC

3501 CGCGGGCCGG TCCCCCCCGC CGGGTCCGCC CCCGGGGCCG CGGTTCCGCG CGCGCCTCGC CTCGGCCGGC GCCTAGCAGC CGACTTAGAA CTGGTGCGGA

3601 CCAGGGGAAT CCGACTGTTT AATTAAAACA AAGCATCGCG AAGGCCCGCG GCGGGTGTTG ACGCGATGTG ATTTCTGCCC AGTGCTCTGA ATGTCAAAGT

3701 GAAGAAATTC AATGAAGCGC GGGTAAACGG CGGGAGTAAC TATGACTCTC TTAAGGTAGC CAAATGCCTC GTCATCTAAT TAGTGACGCG CATGAATGGA

3801 TGAACGAGAT TCCCACTGTC CCTACCTACT ATCCAGCGAA ACCACAGCCA AGGGAACGGG CTTGGCGGAA TCAGCGGGGA AAGAAGACCC TGTTGAGCTT

3901 GACTCTAGTC TGGCACGGTG AAGAGACATG AGAGGTGTAG AATAAGTGGG AGGCCCCCGG CGCCCCCCCG GTGTCCCCGC GAGGGGCCCG GGGCGGGGTC

4001 CGCGGCCCTG CGGGCCGCCG GTGAAATACC ACTACTCTGA TCGTTTTTTC ACTGACCCGG TGAGGCGGGG GGGCGAGCCC GAGGGGCTCT CGCTTCTGGC

4101 GCCAAGCGCC CGCCCGGCCG GGCGCGACCC GCTCCGGGGA CAGTGCCAGG TGGGGAGTTT GACTGGGGCG GTACACCTGT CAAACGGTAA CGCAGGTGTC

**H2 TTTTTTTTTTTTTTTTTTTTTTTTTTTTTTTTTTTTTTTTTTTTTTTTTTCAGG TGGGGAGTTT GACTGGGGCG GTACACCTGT CAAACGGT**

**A3 TTTTTTTTTTTTTTTTTTTTTTTTTTTTTTTTTTTTTTTTTTTTTTTTTTCAGG TGGGGAGTTT GACTGGGGCG GTACACCTGT CAAACGGT**

4201 CTAAGGCGAG CTCAGGGAGG ACAGAAACCT CCCGTGGAGC AGAAGGGCAA AAGCTCGCTT GATCTTGATT TTCAGTACGA ATACAGACCG TGAAAGCGGG

4301 GCCTCACGAT CCTTCTGACC TTTTGGGTTT TAAGCAGGAG GTGTCAGAAA AGTTACCACA GGGATAACTG GCTTGTGGCG GCCAAGCGTT CATAGCGACG

4401 TCGCTTTTTG ATCCTTCGAT GTCGGCTCTT CCTATCATTG TGAAGCAGAA TTCGCCAAGC GTTGGATTGT TCACCCACTA ATAGGGAACG TGAGCTGGGT

4501 TTAGACCGTC GTGAGACAGG TTAGTTTTAC CCTACTGATG ATGTGTTGTT GCCATGGTAA TCCTGCTCAG TACGAGAGGA ACCGCAGGTT CAGACATTTG

4601 GTGTATGTGC TTGGCTGAGG AGCCAATGGG GCGAAGCTAC CATCTGTGGG ATTATGACTG AACGCCTCTA AGTCAGAATC CCGCCCAGGC GAACGATACG

4701 GCAGCGCCGC GGAGCCTCGG TTGGCCTCGG ATAGCCGGTC CCCCGCCTGT CCCCGCCGGC GGGCCGCCCC CCCCTCCACG CGCCCCGCCG CGGGAGGGCG

4801 CGTGCCCCGC CGCGCGCCGG GACCGGGGTC CGGTGCGGAG TGCCCTTCGT CCTGGGAAAC GGGGCGCGGC CGGAAAGGCG GCCGCCCCCT CGCCCGTCAC

4901 GCACCGCACG TTCGTGGGGA ACCTGGCGCT AAACCATTCG TAGACGACCT GCTTCTGGGT CGGGGTTTCG TACGTAGCAG AGCAGCTCCC TCGCTGCGAT

5001 CTATTGAAAG TCAGCCCTCG ACACAAGGGT TTGTC
