# Supplementary material for: Selective Release of MicroRNA Species from Normal and Malignant Mammary Epithelial Cells
Source: PLoS One. 2010 Oct 20;5(10):e13515. doi: 10.1371/journal.pone.0013515 (PMC2958125; doi:10.1371/journal.pone.0013515)
Supplement: Table S3 — Oligonucleotides used for Mature miRNA Quantitation. (0.07 MB DOC) [file pone.0013515.s008.doc]

|  | **primers** |  |  |  |  |  |
| --- | --- | --- | --- | --- | --- | --- |
|  | **for linker ligation only** | **for linker ligation and stem-loops** | **for stem loops only** | **sequence** | **Stem looped primer** |  |
| let-7c |  | hsa-let7c_15 |  | TGAGGTAGTAGGTTG | GTCGTATCCAGTGCAGGGTCCGAGGTATTCGCACTGGATACGACAACCAT | |
| let-7f | hsa-let7f_14 |  |  | TGAGGTAGTAGATTG | N/A |  |
| let-7g |  | hsa-let7g_16 |  | TGAGGTAGTAGTTTGT | GTCGTATCCAGTGCAGGGTCCGAGGTATTCGCACTGGATACGACAACTGT | |
| miR-107 |  | hsa-miR107_15 |  | AGCAGCATTGTACAG | GTCGTATCCAGTGCAGGGTCCGAGGTATTCGCACTGGATACGACtgatag |  |
| miR-1246 |  | hsa-miR1246_16 |  | AATGGATTTTTGGAGC | GTCGTATCCAGTGCAGGGTCCGAGGTATTCGCACTGGATACGACCCTGCT | |
| miR-1275 | hsa-miR-1275_14 |  |  | GTGGGGGAGAGGCT | N/A |  |
| miR-1275 |  |  | hsa-miR-1275_15* | GTGGGGGGAGAGGCT | GTCGTATCCAGTGCAGGGTCCGAGGTATTCGCACTGGATACGACGACAGC | |
| miR-148a |  |  | miR-148a_15 | tcagtgcactacagaa | GTCGTATCCAGTGCAGGGTCCGAGGTATTCGCACTGGATACGACacaaag | |
| miR-149* |  | hsa-miR149*_15 |  | AGGGAGGGACGGGGG | GTCGTATCCAGTGCAGGGTCCGAGGTATTCGCACTGGATACGACCTCCCT | |
| miR-16 |  | hsa-miR16_14 |  | TAGCAGCACGTAAA | GTCGTATCCAGTGCAGGGTCCGAGGTATTCGCACTGGATACGACcgccaa |  |
| miR-1915 | hsa-miR1915_15 |  |  | CCCCAGGGCGACGCG | N/A |  |
| miR-196a |  |  | miR-196a_15 | taggtagtttcatgt | GTCGTATCCAGTGCAGGGTCCGAGGTATTCGCACTGGATACGACcccaac | |
| miR-200b |  |  | miR-200b_15 | taatactgcctggta | GTCGTATCCAGTGCAGGGTCCGAGGTATTCGCACTGGATACGACtcatca | |
| miR-200c | hsa-miR200c_14 |  |  | TAATACTGCCGGGT | N/A |  |
| miR-21 |  | hsa-mir21_15 |  | TAGCTTATCAGACTG | GTCGTATCCAGTGCAGGGTCCGAGGTATTCGCACTGGATACGACtcaaca | |
| miR-210 |  |  | miR-210_15 | ctgtgcgtgtgacag | GTCGTATCCAGTGCAGGGTCCGAGGTATTCGCACTGGATACGACtcagcc | |
| miR-22 | hsa-miR-22_14 |  |  | AAGCTGCCAGTTGA | N/A |  |
| miR-23a |  | hsa-miR23a_14 |  | ATCACATTGCCAGG | GTCGTATCCAGTGCAGGGTCCGAGGTATTCGCACTGGATACGACGGAAAT | |
| miR-27b |  | hsa-miR27b_14-5 |  | CAGTGGCTAAGTTC | N/A |  |
| miR-30c | hsa-miR30c_15 |  |  | TGTAAACATCCTACA | N/A |  |
| miR-335 |  |  | miR-335_15 | tcaagagcaataacg | GTCGTATCCAGTGCAGGGTCCGAGGTATTCGCACTGGATACGACacattt | |
| miR-345 | hsa-miR345_15 |  |  | GCTGACTCCTAGTCC | N/A |  |
| miR-373 |  |  | miR-373_15 | gaagtgcttcgattt | GTCGTATCCAGTGCAGGGTCCGAGGTATTCGCACTGGATACGACacaccc | |
| miR-451 |  | hsa-miR451_16 |  | AAACCGTTACCATTAC | GTCGTATCCAGTGCAGGGTCCGAGGTATTCGCACTGGATACGACAACTCA | |
| miR-483-5P |  | hsa-miR483-5p_15 |  | AAGACGGGAGGAAAG | GTCGTATCCAGTGCAGGGTCCGAGGTATTCGCACTGGATACGACCTCCCT | |
| miR-520c |  |  | miR-520c_15 | aaagtgcttcctttt | GTCGTATCCAGTGCAGGGTCCGAGGTATTCGCACTGGATACGACaccctc | |
| miR-638 | hsa-miR638_15 |  |  | AGGGATCGCGGGCGG | N/A |  |
| miR-720 |  | hsa-miR720_15 |  | TCTCGCTGGGGCCTC | GTCGTATCCAGTGCAGGGTCCGAGGTATTCGCACTGGATACGACTGGAGG | |
| miR-99a |  | hsa-miR100_15 |  | AACCCGTAGATCCGA | GTCGTATCCAGTGCAGGGTCCGAGGTATTCGCACTGGATACGACcacaag | for mouse work, plasma standardization |
| MODBAN | 3'modban_rcrtPCR | |  | ATTGATGGTGCCTACAG | | primer for linkered PCR for rt and PCR |
