# Supplementary material for: Selective Release of MicroRNA Species from Normal and Malignant Mammary Epithelial Cells
Source: PLoS One. 2010 Oct 20;5(10):e13515. doi: 10.1371/journal.pone.0013515 (PMC2958125; doi:10.1371/journal.pone.0013515)
Supplement: Table S4 — Stem Looped Primers used for Simultaneous Analyses of Multiple Transcripts. (0.05 MB DOC) [file pone.0013515.s009.doc]

| STEM-LOOPED PRIMER AMPLIFICATION | |  |  |
| --- | --- | --- | --- |
| hsa- | MIRNA SEQUENCE | PRIMER NAME | PRIMER SEQUENCE |
| miR-16 | UAGCAGCACGUAAAUA*UUGGCG* | hsa-miR-16-14 | TAGCAGCACGTAAA |
|  |  | miR-16_14-loli | GTCGTATCCAGTGCAGGGTCCGAGGTATTCGCACTGGATACGAC*CGCCAA* |
|  |  |  |  |
| miR-1246 | AAUGGAUUUUUGG*AGCAGG* | 1246_loli | GTCGTATCCAGTGCAGGGTCCGAGGTATTCGCACTGGATACGAC*CCTGCT* |
|  |  | 1246_16 | AATGGATTTTTGGAGC |
|  |  |  |  |
| miR-720 | UCUCGCUGGGG*CCUCCA* | 720_loli | GTCGTATCCAGTGCAGGGTCCGAGGTATTCGCACTGGATACGAC*TGGAGG* |
|  |  | 720_14 | CTCGCTGGGGCCTC |
|  |  |  |  |
| miR-451 | AAACCGUUACCAUUAC*UGAGUU* | 451_loli | GTCGTATCCAGTGCAGGGTCCGAGGTATTCGCACTGGATACGAC*AACTCA* |
|  |  | hsa-miR451_15 | AAACCGTTACCATTA |
|  |  |  |  |
| let-7g | UGAGGUAGUAGUUUGU*ACAGUU* | let-7g-loli | GTCGTATCCAGTGCAGGGTCCGAGGTATTCGCACTGGATACGAC*AACTGT* |
|  |  | hsa-let-7g-16 | TGAGGTAGTAGTTTGT |
|  |  |  |  |
| miR-99A | AACCCGUAGAUCCGAU*CUUGUG* | miR-100-loli | GTCGTATCCAGTGCAGGGTCCGAGGTATTCGCACTGGATACGAC*CACAAG* |
| (miR-100) |  | 100-15 | AACCCGTAGATCCGA |
|  |  |  |  |
| miR-210 | CUGUGCGUGUGACAGC*GGCUGA* | miR-210-loli | GTCGTATCCAGTGCAGGGTCCGAGGTATTCGCACTGGATACGAC*TCAGCC* |
|  |  | miR-210_15 | CTGTGCGTGTGACAG |
|  |  |  |  |
| internal loading control: |  |  |  |
| INT-RNA | GGAAAGGGGAACCCCUGUGUGU | INT_RNA-loli | GTCGTATCCAGTGCAGGGTCCGAGGTATTCGCACTGGATACGAC*ACACAC* |
|  |  | INT_15 | GGAAAGGGGAACCCC |
|  |  |  |  |
|  |  | INT_RNA | GGGGGGAAAAACCCCUUUUUCC |
|  |  |  |  |
| miR-1275 | GUGGGGGAGAG*GCUGUC* | 1275_loli | GTCGTATCCAGTGCAGGGTCCGAGGTATTCGCACTGGATACGAC*GACAGC* |
|  |  | hsa-miR-1275_15* | GTGGGGGGAGAGGCT |
|  |  |  |  |
| lolirev |  | loli_rev | GTGCAGGGTCCGAGGT |
