# Supplementary material for: Selective Release of MicroRNA Species from Normal and Malignant Mammary Epithelial Cells
Source: PLoS One. 2010 Oct 20;5(10):e13515. doi: 10.1371/journal.pone.0013515 (PMC2958125; doi:10.1371/journal.pone.0013515)
Supplement: Table S5 — Confirmed sequences of qPCR products quantified using linker ligated primers. (0.03 MB DOC) [file pone.0013515.s010.doc]

**hsa-miR-221 AGCUACAUUGUCUGCTGGGUUUC**

1_B05_271599_ECRC AGCTACATTGTCTGCTGGGTTTC

1_F03_271602_CCRC AGCTACATTGTCTGCTGGGTTTC

1_B04_271597_EA AGCTACATTGTCTGCTGGGTTTC

1_F02_271600_CA AGCTACATTGTCTGCTGGGTT**A**C

1_F04_271598_EBRC AGCTACATTGTCTGCTGGGTTTC

**hsa-miR-222 AGCUACAUCUGGCUACUGGGU**

1_C04_271603_EA AGCTACATCTGGCTACTGGGT

1_G04_271604_EB AGCTACATCTGGCTACTGGGT

1_G02_271606_CA AGCTACATCTGGCTACTGGGT

1_C03_271607_CB AGCTACATCTGGCTACTGGGT

1_G03_271608_CC AGCTACATCTGGCTACTGGGT

**hsa-miR-100 AACCCGUAGAUCCGAACUUGUG**

**hsa-miR-99a AACCCGUAGAUCCGAUCUUGUG**

1_A04_271609_EA AACCCGTAGATCCGA**T**CTTGT

1_E03_271614_CC AACCCGTAGATCCGA**T**CTTGTG

1_E02_271612_CA AACCCGTAGATCCGA**T**CTTGTG

1_A03_271613_CB AACCCGTAGATCCGA**T**CTTGT

1_A05_271611_EC AACCCGTAGATCCGA**T**CTTGTG

**hsa-miR-720 UCUCGCUGGGGCCUCCA**

1_D04_271615_EA T**A**TCGCTGGGGCCTCCA

1_D05_271617_EC TCTCGCTGGGGCCTCCA

1_H04_271616_EB TCTCGCTGGGGCCTCCA

1_D03_271619_CB TCTCGCTGGGGCCTCCA

1_H02_271618_CArc TCTCGCTGGGGCCTCCA

**Confirmed sequences for looped miRs**

**hsa-miR-16 UAGCAGCACGUAAAUAUUGGCG**

1_A09_2742 TAGCAGCACGTAAATATTGGCG

1_B09_2742 TAGCAGCACGTAAATATTGGCG

1_H11_2742 TAGCAGCACGTAAATATTGGCG

**hsa-miR-1246 AAUGGAUUUUUGGAGCAGG**

1_E07_2742rc AATGGATTTTTGGAGCAGG

1_F07_2742rc AATGGATTTTTGGAGCAGG

1_G07_2742rc AATGGATTTTTGGAGCAGG

1_H07_2742 AATGGATTTTTGGAGCAGG

**hsa-miR-720 UCUCGCUGGGGCCUCCA**

1_H10_2742 TCTCGCTGGGGCCTCCA

1_B11_2742 TCTCGCTGGGGCCTCCA

1_C11_2742 TCTCGCTGGGGCCTCCA

**hsa-miR-451 AAACCGUUACCAUUACUGAGUU**

1_B10_2742 rc AAACCGTTACCATTACTGAG--

1_A10_2742 rc AAACCGTTACCATTACTGAGTT

1_C10_2742 AAACCGTTACCATTACTGAGTT

**hsa-let-7g UGAGGUAGUAGUUUGUACAGUU**

1_D11_2742 TGAGGTAGTAGTTTGTACAGTT

1_F11_2742 TGAGGTAGTAGTTTGTACAGTT

**>hsa-miR-1275 GUGGGGGAGAGGCUGUC**

1_E08_2742 Trc GTGGGGGAGAGGCTGTC

1_C08_2742 Lrc GTGGGGGAGAGGCTGTC

1_H08_2742 U GTGGGGGAGAGGCTGTC

1_A08_2742 L GTGGGGGAGAGGCTGA-
