# Supplementary material for: Selective Release of MicroRNA Species from Normal and Malignant Mammary Epithelial Cells
Source: PLoS One. 2010 Oct 20;5(10):e13515. doi: 10.1371/journal.pone.0013515 (PMC2958125; doi:10.1371/journal.pone.0013515)
Supplement: Table S6 — Primers for Quantifying pre-miRNAs and Other Immature miRNAs. (0.07 MB DOC) [file pone.0013515.s011.doc]

| RNA |  | name | sequence |
| --- | --- | --- | --- |
| pre-let-7f | 5' | let7Fm-1Fm | GATTGTATAGTTGTGGGGTAGTG |
| pre-let-7f | 3' | let7Rm-1Rm | GGGAAGGCAATAGATTGTATAG |
| pre-let-7g | 5' | let7gFm | GTAGTAGTTTGTACAGTTTGAGGGT |
| pre-let-7g | 3' | let7gRm | GGCAGTGGCCTGTACAGT |
| pre-let-7c | 5' | let7cFm | TTGAGGTAGTAGGTTGTATGGTT |
| pre-let-7c | 3' | let7cRm | GGAAAGCTAGAAGGTTGTACAG |
| pre-let-7c | pri-S | Let-7c-US1 | TGAAGCAACATTGGAAGC |
| pre-let-7c | pri-L | Let-7c-US2 | TCTATATCCTTGCCAAGCC |
| pre-miR-100 | 5' | miR-100Fm | AACCCGTAGATCCGAACTTG |
| pre-miR-100 | 3' | miR-100Rm | TACCTATAGATACAAGCTTGTGCG |
| pre-miR-1246 | 5' | hsa-miR1246_19 | TTGAATGGATTTTTGGAGC |
| pre-miR-1246 | 3' | 1246_matrev_22 | ATTGCTAGCCTATGGATTGATT |
| pre-miR-1275 | 5' | hsa-miR-1275_15* | GTGGGGGGAGAGGCT |
| pre-miR-1275 | 3' | 1275_mat rev | TCCCTCTGCCTTGGG |
| pre-miR-149* | 5' | 149*_matfor | GCCGGCGCCCGAGCT |
| pre-miR-149* | 3' | 149*_matrev | GCCCCCGTCCCTCCC |
| pre-miR-155 | 5' | miR-155Fm | GTTAATGCTAATCGTGATAGGG |
| pre-miR-155 | 3' | miR-155Rm | GCTAATATGTAGGAGTCAGTTGGA |
| pre-miR-16 | 5' | miR-016-1Fmm | GCAGCACGTAAATATTGGCGT |
| pre-miR-16 | 3' | miR-016-1Rm | CAGCAGCACAGTTAATACTGGAGA |
| pre-miR-1915 | 5' | 1915_matfor | TGAGAGGCCGCACCT |
| pre-miR-1915 | 3' | 1915_matrev_13 | CGCGTCGCCCTGG |
| pre-miR-200c | 5' | miR-200cFm | CTCGTCTTACCCAGCAGTGT |
| pre-miR-200c | 3' | miR-200cRm2 | CATCATTACCCGGCAGTATTAG |
| pre-miR-200c | pri-S | miR-200c US1 | GAAGCTGCCTGACCCAAG |
| pre-miR-200c | pri-L | miR-200c US2 | CAGGGATCTGCAGCTTTTC |
| pre-miR-221 | 5' | miR-221Fm | CCTGGCATACAATGTAGATTTCTG |
| pre-miR-221 | 3' | miR-221Rm | AAACCCAGCAGACAATGTAGCT |
| pre-miR-221 | pri-S | miR-221 US1 | AAATAGTATGTGAGAATTACTTGCAAGC |
| pre-miR-221 | pri-L | miR-221 US2 | GAAATTTTGTTGGTAGTAGGTAAGTCC |
| pre-miR-222 | 5' | miR-222(p)Fm | CCCCAGAAGGCAAAGGAT |
| pre-miR-222 | 3' | miR-222(p)Rm | CTCTCTCAGGACACTGAAGCAG |
| pre-miR-23a | 5' | miR-023aFm | CTGGGGTTCCTGGGGAT |
| pre-miR-23a | 3' | miR-023aRm | TGGTAATCCCTGGCAATGTG |
| pre-miR-27 | 5' | miR-027a,bFm | GCAGGGCTTAGCTGCTTG |
| pre-miR-27 | 3' | miR-027a,bRm | GGCGGAACTTAGCCACTGT |
| pre-miR-30c | 5' | miR-030c-1,2Fm | TGTGTAAACATCCTACACTCTCAG |
| pre-miR-30c | 3' | miR-030c-1,2Rm | GAGTAAACAACCCTCTCCCA |
| pre-miR-451 | 5' | hsa-miR-451_21 | CAAGGAAACCGTTACCATTAC |
| pre-miR-451 | 3' | 451_mat rev | TCTGGGTATAGCAAGAGAACC |
| pre-miR-638 | 5' | 638_13 | AGGGATCGCGGGC |
| pre-miR-638 | 3' | 638_matrev | AGCGCCGCAGTTACG |
| pre-pre-miR-21 | 5' | miR-021Fm | GCTTATCAGACTGATGTTGACTG |
| pre-pre-miR-21 | 3' | miR-021Rm | CAGCCCATCGACTGGTG |
| pre-pre-miR-21 | pri-S | miR-021 US1 | ATCTCCATGGCTGTACCACC |
| pre-pre-miR-21 | pri-L | miR-021 US2 | TCAAATCCTGCCTGACTGTC |
