# Supplementary material for: Selective Release of MicroRNA Species from Normal and Malignant Mammary Epithelial Cells
Source: PLoS One. 2010 Oct 20;5(10):e13515. doi: 10.1371/journal.pone.0013515 (PMC2958125; doi:10.1371/journal.pone.0013515)
Supplement: Table S7 — Sequences of Immature miRNAs Detected. (0.05 MB DOC) [file pone.0013515.s012.doc]

1 110

**hsa-mir-221**UGAACAUCCA GGUCUGGGGC AUGAA*CC*U*GG* *CA*U*ACAA*U*G*U *AGA*UUU*C*U*G*U *G*UU*CG*UU*AGG* *CAACAGC*U*AC* *A*UU*G*U*C*U*GC*U *GGG*UUUCAGG CUACCUGGAA ACAUGUUCUC

**C**mim221C_1 *CCTGG* *CATACAATGT* *AGATTTCTGT* *GTTCGTTAGG* *CAACAGCTAC* *ATTGTCTGCT* *GGGTTT*

**C**mim221B_1 *CCTGG* *CATACAATGT* *AGATTTCTGT* *GTTCGTTAGG* *CAACAGCTAC* *ATTGTCTGCT* *GGGTTT*

**E**mrcm221C_ *CCTGG* *CATACAATGT* *AGATTTCTGT* *GTTCGTTAGG* *CAACAGCTAC* *ATTGTCTGCT* *GGGTTT*

**C**rcim221A_ *CCTGG* *CATACAATGT* *AGATTTCTGT* *GTTCGTTAGG* *CAACAGCTAC* *ATTGTCTGCT* *GGGTTT*

**E**mim221A_1 *CCTGG* *CATACAATGT* *AGATTTCTGT* *GTTCGTTAGG* *CAACAGCTAC* *ATTGTCTGCT* *GGGTTT*

**E**mim221B_1 CGC*GT* CGC*C*C*T*GG*G*C AG*A*A*A*T*CTAC* *ATTGT*A*TGC*C A*GG*

1 72

**hsa-mir-21** UGUCGGGUA*G* *C*UU*A*U*CAGAC* U*GA*U*G*UU*GAC* U*G*UU*GAA*U*C*U *CA*U*GGCAACA* *CCAG*U*CGA*U*G* *GGC*U*G*UCUGA CA

**C**mim21B_16 *G* *CTTATCAGAC* *TGATGTTGAC* *TGTTGAATCT* *CATGGCAACA* *CCAGTCGATG* *GGCTG*

**C**rcim21C_1 *G* *CTTATCAGAC* *TGATGTTGAC* *TGTTGAATCT* *CATGGCAACA* *CCAGTCGATG* *GGCTG*

**E**rcim21B_1 *G* *CTTATCAGAC* *TGATGTTGAC* *TGTTGAATCT* *CATGGCAACA* *CCAGTCGATG* *GGCTG*

**C**rcim21A_1 *G* *CTTATCAGAC* *TGATGTTGAC* *TGTTGAATCT* *CATGGCAACA* *CCAGTCGATG* *GGCTG*

**E**rcim21C_1 *G* *CTTATCAGAC* *TGATGTTGAC* *TGTTGAATCT* *CATGGCAACA* *CCAGTCGATG* *GGCTG*

**E**rcim21A_1 *G* *CTTATCAGAC* *TGATGTTGAC* *TGTTGAATCT* *CATGGCAACA* *CCAGTCGATG* *GGCTG*

1 73

**hsa-miR-23a** GGCCGGCUG GGGUUCCUGG GGAUGGGAUU UGCUUCCUGU CACAAAUCAC AUUGCCAGGG AUUUCCAACC GACC

**C**C23a_186 CTG GGGTTCCTGG GGATGGGATT TGCTTCCTGT CACAAATCAC ATTGCCAGGG ATTACCA

**E**C23a_183 CTG GGGTTCCTGG GGATGGGATT TGCTTCCTGT CACAAATCAC ATTGCCAGGG ATTACCA

**E**B23a_182 CTG GGGTTCCTGG GGATGGGATT TGCTTCCTGT CACAAATCAC ATTGCCAGGG ATTACCA

**E**A23a_181 CTG GGGTTCCTGG GGATGGGATT TGCTTCCTGT CACAAATCAC ATTGCCAGGG ATTACCA

**C**B23a_185 CTG GGGTTCCTGG GGATGGGATT TGCTTCCTGT CACAAATCAC ATTGCCAGGG ATTACCA

**C**A23a_184 CTG GGGTTCCTGG GGATGGGATT TGCTTCCTGT CACAAATCAC ATTGCCAGGG ATTACCA

1 90

**hsa-mir-161** GUCAGCAGUG CCUU*AGCAGC* *ACG*UA*AA*U*A*U U*GG*C*G*UU*AA*G AUUCU*AA*AAU U*A*U*C*U*CCA*GU *A*UUA*AC*U*G*U*G C*U*GC*UGAAGU AAGGUUGAC

**C**rcim16C_1 *GCAGC* *ACGT*A*AATA*T *TGG*C*G*T*TAA*G A*TT*CT*AA*AA*T TA*T*C*T*CCA*G*T ATT*A*AC*T*GTG CTGC*TG

**C**fcim16B_1* *GCAGC* *ACGT*A*AATA*T *TGG*C*G*T*TAA*G A*TT*CT*AA*AA*T TA*T*C*T*CCA*G*T ATT*A*AC*T*GTG CTGC*TG

**E**fcim16A_1* *GCAGC* *ACGT*A*AATA*T *TGG*C*G*T*TAA*G A*TT*CT*AA*AA*T TA*T*C*T*CCA*G*T ATT*A*AC*T*GTG CTGC*TG

**E**fcim16B_1* *GCAGC* *ACGT*A*AATA*T *TGG*C*G*T*TAA*G A*TT*CT*AA*AA*T TA*T*C*T*CCA*G*T ATT*A*AC*T*GTG CTGC*TG

**C**fcim16A_1* *GCAGC* *ACGT*A*AATA*T *TGG*C*G*T*TAA*G A*TT*CT*AA*AA*T TA*T*C*T*CCA*G*T ATT*A*AC*T*GTG CTGC*TG

**miR-200c**

immature CAGGGAUCU GCAGCUUUUC CGCAGGGAUC CUGGGCCUGA AGCUGCCUGA CCCAAGGUGG GCGGGCUGGG CGGGGGCCCU CGUCUUACCC AGCAGUGUUU GGGUGCGGUU GGGAGUCUCU AAUACUGCCG GGUAAUGAUG GAGG

**E**fiUS2C CAGGGATCT GCAGCTTTTC CGCAGGGATC CTGGGCCTGA AGCTGCCTGA CCCAAGGTGG GCGGGCTGGG CGGGGGCCCT CGTCTTACCC AGCAGTGTTT GGGTGCGGTT GGGAGTCTCT AATACTGCCT GGTAATGATG AC

**E**riUS2B CAGGGATCT GCAGCTTTTC CGCAGGGATC CTGGGCCTGA AGCTGCCTGA CCCAAGGTGG GCGGGCTGGG CGGGGGCCCT CGTCTTACCC AGCAGTGTTT GGGTGCGGTT GGGAGTCTCT AATACTGCCT GGTAATGATG AC

**C**riUS1B TGA AGCTGCCTGA CCCAAGGTGG GCGGGCTGGG CGGGGGCCCT CGTCTTACCC AGCAGTGTTT GGGTGCGGTT GGGAGTCTCT AATACTGCCT GGTAATGATG AC

**C**fiUS1C GA AGCTGCCTGA CCCAAGGTGG GCGGGCTGGG CGGGGGCCCT CGTCTTACCC AGCAGTGTTT GGGTGCGGTT GGGAGTCTCT AATACTGCCT GGTAATGATG AC

**C**fiUS1A GA AGCTGCCTGA CCCAAGGTGG GCGGGCTGGG CGGGGGCCCT CGTCTTACCC AGCAGTGTTT GGGTGCGGTT GGGAGTCTCT AATACTGCCT GGTAATGATG AC

**C**ri200cC CT CGTCTTACCC AGCAGTGTTT GGGTGCGGTT GGGAGTCTCT AATACTGCCT GGTAATGATG AC

**C**ri200cA CT CGTCTTACCC AGCAGTGTTT GGGTGCGGTT GGGAGTCTCT AATACTGCCT GGTAATGATG AC

**E**fi200cA_ CT CGTCTTACCC AGCAGTGTTT GGGTGCGGTT GGGAGTCTCT AATACTGCCT GGTAATGATG AC

**E**fi200cB_ CT CGACTTACCC AGCAGTGTTT GGGTGCGGTT GGGAGTCTCT AATACTGCCT GGTAATGATG AC

1 84

**hsa-let-7g** AGGCUGAG*G*U *AG*U*AG*UUU*G*U *ACAG*UUU*GAG* *GG*U*C*U*A*U*GA*U *ACCACCCGG*U *ACAGGAGA*U*A* *AC*U*G*U*ACAGG* *CCAC*U*GCC*UU GCCA

**C**rcim7gC_1 *GT* *AGTAGTTTGT* *ACAGTTTGAG* *GGTCTATGAT* *ACCACCCGGT* *ACAGGAGATA* *ACTGTACAGG* *CCACTGCC*

**C**rcim7gB_1 *GT* *AGTAGTTTGT* *ACAGTTTGAG* *GGTCTATGAT* *ACCACCCGGT* *ACAGGAGATA* *ACTGTACAGG* *CCACTGCC*

**E**mim7gA_15 *GT* *AGTAGTTTGT* *ACAGTTTGAG* *GGTCTATGAT* *ACCACCCGGT* *ACAGGAGATA* *ACTGTACAGG* *CCACTGCC*

**C**mim7gA_16 *GT* *AGTAGTTTGT* *ACAGTTTGAG* *GGTCTATGAT* *ACCACCCGGT* *ACAGGAGATA* *ACTGTACAGG* *CCACTGCC*
